# Spontaneous reactivation of latent HIV-1 promoters is linked to the cell cycle as revealed by a genetic-insulators-containing dual-fluorescence HIV-1-based vector

**DOI:** 10.1038/s41598-018-28161-y

**Published:** 2018-07-05

**Authors:** Yik Lim Kok, Stefan Schmutz, Anne Inderbitzin, Kathrin Neumann, Audrey Kelley, Lisa Jörimann, Mohaned Shilaih, Valentina Vongrad, Roger D. Kouyos, Huldrych F. Günthard, Christian Berens, Karin J. Metzner

**Affiliations:** 1 Division of Infectious Diseases and Hospital Epidemiology, University Hospital Zurich, Zurich, Switzerland; 2 Institute of Medical Virology, University of Zurich, Zurich, Switzerland; 3 Life Science Zurich Graduate School, University of Zurich, Zurich, Switzerland; 4 Institute of Molecular Pathogenesis, Friedrich-Loeffler-Institut, Jena, Germany

## Abstract

Long-lived latently HIV-1-infected cells represent a barrier to cure. We developed a dual-fluorescence HIV-1-based vector containing a pair of genetic insulators flanking a constitutive fluorescent reporter gene to study HIV-1 latency. The protective effects of these genetic insulators are demonstrated through long-term (up to 394 days) stable fluorescence profiles in transduced SUP-T1 cells. Analysis of 1,941 vector integration sites confirmed reproduction of HIV-1 integration patterns. We sorted monoclonal cells representing latent HIV-1 infections and found that both vector integration sites and integrity of the vector genomes influence the reactivation potentials of latent HIV-1 promoters. Interestingly, some latent monoclonal cells exhibited a small cell subpopulation with a spontaneously reactivated HIV-1 promoter. Higher expression levels of genes involved in cell cycle progression are observed in these cell subpopulations compared to their counterparts with HIV-1 promoters that remained latent. Consistently, larger fractions of spontaneously reactivated cells are in the S and G2 phases of the cell cycle. Furthermore, genistein and nocodazole treatments of these cell clones, which halted cells in the G2 phase, resulted in a 1.4–2.9-fold increase in spontaneous reactivation. Taken together, our HIV-1 latency model reveals that the spontaneous reactivation of latent HIV-1 promoters is linked to the cell cycle.

## Introduction

Upon entry into a CD4^+^ T cell, the human immunodeficiency virus type 1 (HIV-1) integrates its reverse-transcribed viral DNA into the host’s genome^1^. The integrated provirus has two fates: it either continues its replication cycle to produce progeny virions or remains latent in the host cell^1^. The latent HIV-1 reservoir is unsusceptible to both the host individual’s immune system and antiretroviral therapy (ART), which is currently only effective against active infections^2^. More importantly, ART cessation leads to rebound of HIV-1, thus necessitating lifelong therapy^3^.

Studies examining features driving the establishment and maintenance of HIV-1 latency have been limited by the low frequencies of cells latently infected with replication-competent HIV-1 in patients (~1–10^2^ per 10^6^ CD4^+^ T cells)^4,5^ and the lack of phenotypic markers to identify these cells^6^. To circumvent these obstacles, *in vitro* models were developed to recapitulate HIV-1 infection and latency. Earlier models used HIV-1-based vectors encoding one fluorescent reporter gene to transduce and subsequently identify cells harbouring an active or latent HIV-1 promoter, *i.e*., those which expressed the fluorescent reporter gene constitutively or only when activated externally, respectively^7–9^. Dual-fluorescence vectors were later developed to identify cells harbouring a latent HIV-1 promoter without the need for external activation^10–12^. These second generation vectors have an additional fluorescent reporter gene whose expression is driven by a constitutive promoter to identify transduced cells irrespective of their HIV-1 promoter activity.

These models have proved to be valuable in understanding molecular features of HIV-1 latency. For instance, it has been shown that vector integration sites have an influence on the HIV-1 promoter activity^7,13,14^, and Dahabieh *et al*. showed that the HIV-1 promoter activity correlates with the degree of activation of cells during transduction^11^. Sherrill-Mix *et al*. recently showed that the association between HIV-1 latency and chromosomal position is model dependent^15^. This is likely a consequence of the heterogeneous nature of the latent HIV-1 reservoir^16^, thus arguing for novel models to complement existing ones in order to unravel key determinants of HIV-1 latency-associated events.

**Figure 1:**
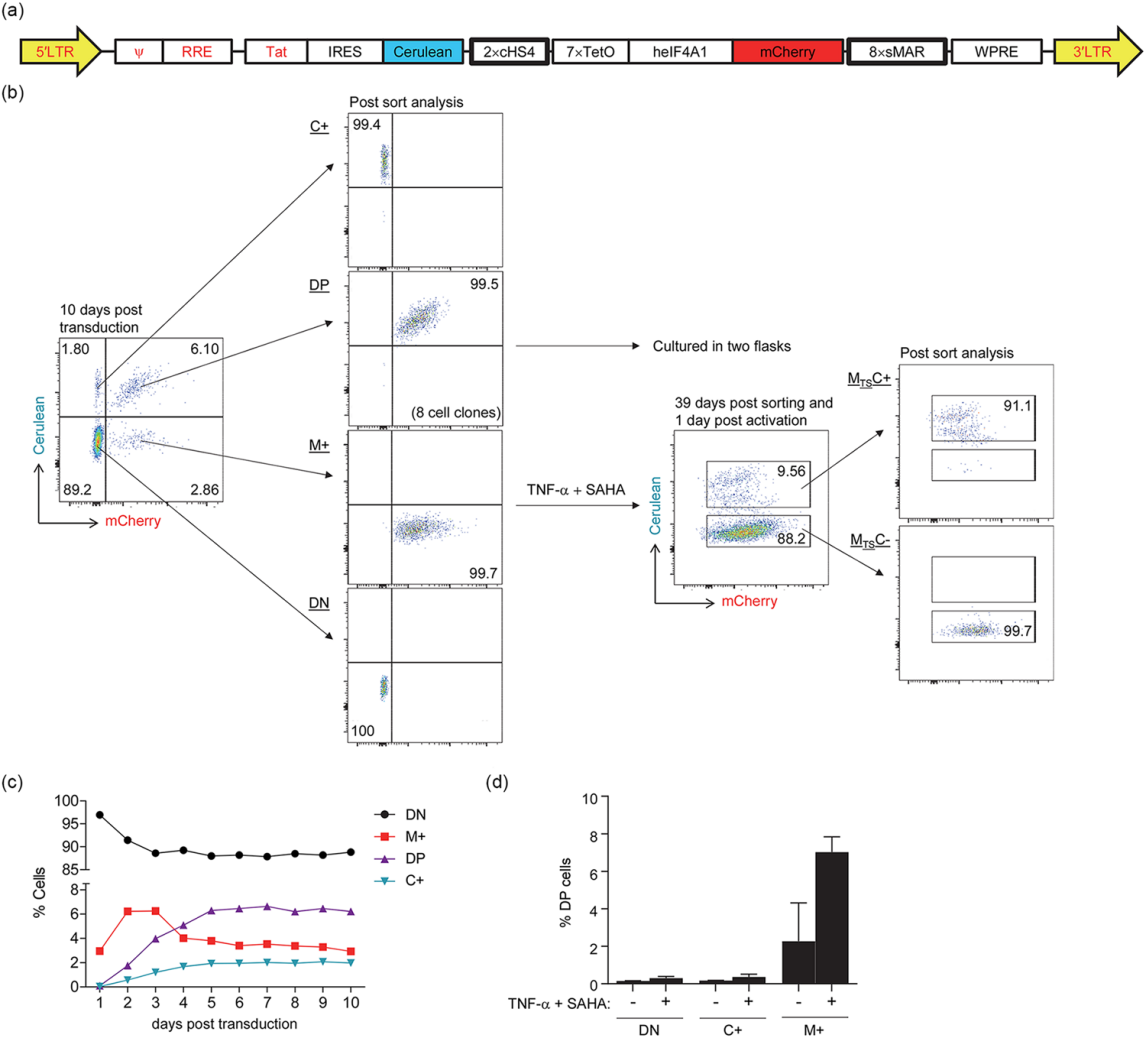
Transduction of SUP-T1 cells with LTatC[M], a dual-fluorescence HIV-1-based vector. (**a**) Schematic diagram of LTatC[M]. The expression of Cerulean is driven by HIV-1 5′ long terminal repeat (LTR) and supported by HIV-1 transactivator of transcription (Tat) via a positive feedback loop. The expression of mCherry is driven by the constitutive human elongation initiation factor 4A1 (*heIF4A1*) promoter and the entire cassette is protected from position-effect variegation by a pair of genetic insulators: two copies of the chicken hypersensitive site 4 core (cHS4) and eight copies of synthetic matrix attachment region (sMAR). ψ: HIV-1 packaging signal; RRE; HIV-1 Rev response element; IRES: Internal ribosomal entry site; TetO: Tet operator; WPRE: Woodchuck hepatitis virus post-transcriptional regulatory element. (**b**) Flow cytometric sorting strategy and purity of transduced SUP-T1 cell populations. SUP-T1 cells transduced with LTatC[M] were sorted 10 days post transduction into four cell populations: single Cerulean positive (C+), double positive (DP), single mCherry positive (M+), and double negative (DN). DP cells were divided equally and cultured in two flasks. A portion of M+ cells was expanded in culture for 39 days and then activated with TNF-α and SAHA for one day prior to sorting into cell populations that became Cerulean^+^ (M_TS_C+) and that remained Cerulean^−^ (M_TS_C−). (**c**) Fluorescence profiles of the four cell populations that arose from transduction of SUP-T1 cells with LTatC[M]: DN, M+, DP, and C+, were measured with flow cytometry daily for 10 days. Each datapoint represents the mean of two independent transductions (n = 2) and error bars depict standard error means. Some error bars are within datapoints. (**d**) DN, C+, and M+ cell populations sorted from LTatC[M]-tranduced SUP-T1 cells were treated with TNF-α and SAHA for one day and the induction of DP cells from these cell populations was measured with flow cytometry. In the case of DN cell population, the induction of C+ and M+ cells was also measured but not distinguished from DP cell induction. The means of two independent measurements (n = 2) are indicated by the heights of the bars and standard error means are indicated by the error bars.

We have developed a novel HIV-1-based vector (LTatC[M]) consisting of two fluorescent reporter gene cassettes: Cerulean and mCherry (Fig. 1a). The expression of Cerulean is driven by the HIV-1 promoter in the viral 5′ long terminal repeat (5′ LTR) and supported by the HIV-1 transactivator of transcription (Tat) via a positive feedback loop whereas the expression of mCherry is driven by the constitutive human elongation initiation factor 4A1 (*heIF4A1*) promoter (Fig. 1a). As such, Cerulean reports the activity of the HIV-1 promoter (active or latent) and mCherry labels transduced cells, enabling the identification of cells harbouring an active HIV-1 promoter and simultaneously distinguishing cells harbouring a latent HIV-1 promoter from untransduced cells. Additionally, the mCherry cassette is flanked by a pair of genetic insulators, the chicken hypersensitive site 4 core (cHS4) and synthetic matrix attachment region (sMAR) (Fig. 1a), to prevent (i) transcriptional interference between Cerulean and mCherry^17–19^ that might lead to artefactual HIV-1 latency and (ii) silencing of mCherry due to position-effect variegation^20–25^, thus ensuring its long-term constitutive expression.

In the present study, we first characterized our vector LTatC[M] and verified its capacity to reproduce features of active and latent HIV-1 infections. We then transduced SUP-T1 cells with LTatC[M] pseudotyped with vesicular stomatitis virus glycoprotein G (VSV-G) and subsequently sorted out bulk cell populations, as well as monoclonal cells, representing active and latent HIV-1 infections for further analysis. With our model, we observed that some monoclonal cells constantly exhibited a spontaneously reactivated HIV-1 promoter and found that this phenomenon is linked to the cell cycle.

## Results

### Transduction of SUP-T1 cells with LTatC[M] yields active and latent HIV-1 phenotypes

Transduction of SUP-T1 cells with VSV-G-pseudotyped LTatC[M] (Fig. 1a) yielded four cell populations: double positive (DP), single Cerulean positive (C+), single mCherry positive (M+), and double negative (DN) (Fig. 1b). The HIV-1 promoter in DP and C+ cells was active since Cerulean was expressed, and thus they represented active HIV-1 infections. Correspondingly, latent HIV-1 infections were represented by M+ cells due to their lack of Cerulean expression (Fig. 1b). Henceforth, DP and C+ will be called active and M+ will be called latent to denote the state of the HIV-1 promoter harboured in these cells and not of the host cells.

As we were interested in cell populations with stable phenotypes representing active and latent HIV-1 infections, we examined the kinetics of the emergence of the four cell populations: DP, C+, M+, and DN, for 10 days post transduction (Fig. 1c; Supplementary Fig. S1). The majority of transduced cells appeared to be latent up to 3 days post transduction, although this phenomenon was transient, and a stable fluorescence profile for all transduced cells was established only at 5 days post transduction. The initial fluctuations in fluorescence could be a result of differential temporal regulation of gene expression post integration at different vector integration sites and spontaneous reactivation of latent HIV-1 promoters in M+ cells. Similar kinetics were observed between transduction efficiencies of 5% and 15% (Fig. 1c; Supplementary Fig. S1). Therefore, we sorted with flow cytometry all four cell populations at 10 days post transduction with a purity of >99% for subsequent analyses (Fig. 1b).

Next, DN, C+, and M+ cell populations sorted from LTatC[M]-transduced SUP-T1 cells were treated with TNF-α and SAHA for 24 hours and the induction of DP cells from these cell populations was measured with flow cytometry. Approximately 7% of M+ cells expressed Cerulean and mCherry after induction with TNF-α and SAHA (Fig. 1d), showing that latent M+ cells could be activated to become double positive. The expression of either Cerulean or mCherry in DN cells and mCherry in C+ cells could not be induced with TNF-α and SAHA (Fig. 1d).

### The genetic insulators, cHS4 and sMAR, confer long-term stability of fluorescence profiles in LTatC[M]-transduced SUP-T1 cells

**Figure 2:**
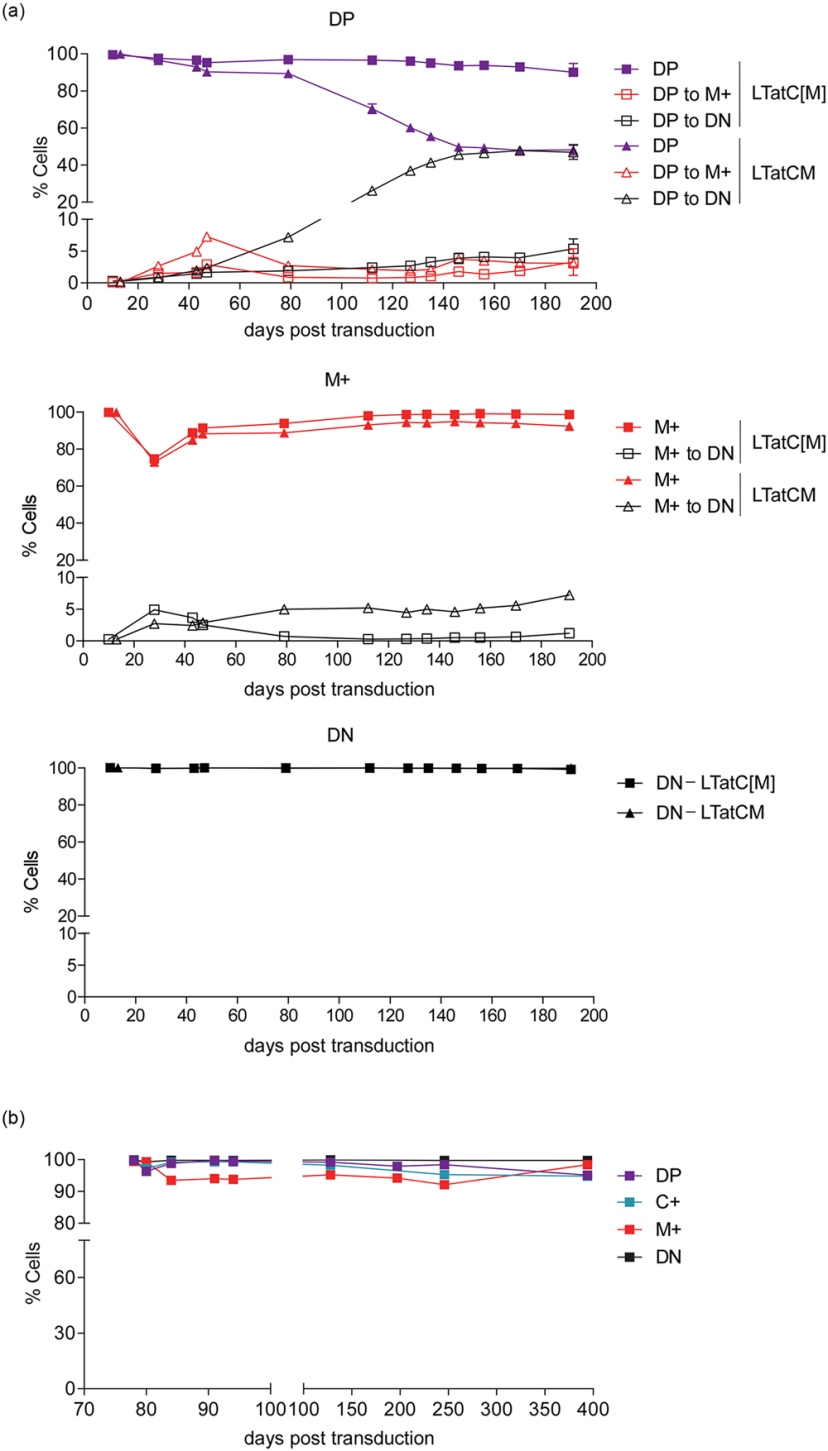
Longitudinal fluorescence profiles of transduced SUP-T1 cell populations. (**a**) SUP-T1 cells were transduced with LTatC[M] (squares) or a vector variant with no genetic insulators in the mCherry cassette (LTatCM) (triangles). Double positive (DP), single mCherry positive (M+), and double negative (DN) cell populations were sorted 10 days post transduction and their fluorescence profiles were measured with flow cytometry for 191 days post transduction. Shaded symbols depict percentages of cells with the fluorescence initially sorted for whereas open symbols depict percentages of cells that became DN and M+. Each datapoint of the DP data set represents the mean of duplicate cultures (n = 2) and error bars depict standard error means. Some error bars are within datapoints. (**b**) Longitudinal fluorescence profiles of sorted SUP-T1 cell populations up to 394 days post transduction in a second transduction experiment.

The short-term kinetics and ratio of active (DP and C+) to latent cells (M+) were comparable between LTatC[M] and a vector variant with no genetic insulators flanking the mCherry cassette (LTatCM) (Supplementary Fig. S1), demonstrating that the genetic insulators did not influence the emergence of latent cells. As the present study focused on the long-term maintenance and persistence of HIV-1 latency, we examined whether the genetic insulators were capable of protecting the mCherry cassette from being silenced over time by monitoring the fluorescence profiles of the sorted DP, M+, and DN cell populations transduced with LTatC[M] or LTatCM up to 191 days post transduction. Over 90% of cell populations transduced with LTatC[M] retained their initial fluorescence profiles for the entire period analysed (Fig. 2). In contrast, mCherry expression in DP cells transduced with LTatCM decreased gradually down to 48% at 191 days post transduction (Fig. 2a). The decrease in mCherry expression in LTatCM-transduced M+ cells was less drastic, although it remained consistently lower by ~5% compared to the LTatC[M]-transduced M+ cells (Fig. 2a). Our results were reproducible in a second transduction of SUP-T1 cells with LTatC[M]. Consistently, over 90% of all sorted cell populations in the second transduction retained their initial fluorescence profiles analysed for 394 days post transduction (Fig. 2b). Therefore, the pair of genetic insulators, cHS4 and sMAR, flanking the mCherry cassette confer long-term protection to mCherry expression from silencing.

### LTatC[M] reproduces HIV-1 integration site patterns

**Figure 3:**
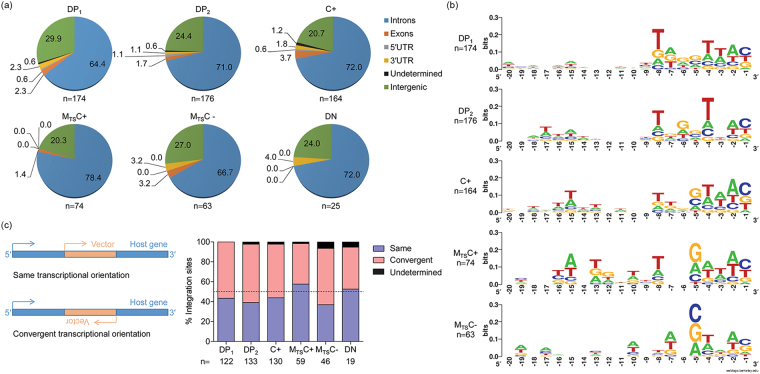
Integration site patterns of LTatC[M] in various SUP-T1 cell populations. (**a**) Genomic distributions of integrated LTatC[M]. (**b**) Consensus sequences of 20 nucleotides upstream of 5′LTR. Double negative (DN) is not shown due to low number of integration sites. (**c**) Transcription orientations of intragenic LTatC[M] relative to its host genes. DP_1_: double positive replicate 1; DP_2_: double positive replicate 2; C+: single Cerulean; M_TS_C+: TNF-α and SAHA-responsive single mCherry positive; M_TS_C−: TNF-α and SAHA-non-responsive single mCherry positive.

To ensure that the genetic-insulators-containing LTatC[M] reproduced integration site patterns of HIV-1, vector integration sites from the sorted cell populations, DP, C+, M_TS_C+, M_TS_C−, and DN, were sequenced and mapped with the Integration Site Analysis Pipeline (InStAP)^26^. M_TS_C+ and M_TS_C− cells were sorted from latent M+ cells in which Cerulean expression was inducible and non-inducible with TNF-α and SAHA, respectively (Fig. 1b). A total of 676 unique LTatC[M] integration sites were analysed. Across all cell populations, the majority of vector integration sites were found in transcription units, although slightly more in the inducible M_TS_C+ cells (Fig. 3a), and in a convergent transcriptional orientation relative to vector-hosting genes, except for M_TS_C+ cells (Fig. 3c). The HIV-1 signature weakly conserved palindromic sequence was also observed at vector integration sites for all cell populations (Fig. 3b). These features were reproducible in a second transduction experiment in which a total of 1,265 unique vector integration sites were analysed (Supplementary Fig. S2).

### LTatC[M] integration site selection and genome integrity influence reactivation potentials of HIV-1 promoters in TNF-α and SAHA-responsive (M_TS_C+) and -non-responsive (M_TS_C−) single mCherry positive cell clones

The influence of HIV-1 integration sites on the reactivation potentials of latent HIV-1 promoters has been demonstrated previously^7,13,14^. To further explore this in our model, we sorted from latent cell populations (M+) from two transductions: 22 latent cell clones in which Cerulean expression was inducible with TNF-α and SAHA (M_TS_C+) and 18 latent cell clones in which Cerulean expression was non-inducible with TNF-α and SAHA (M_TS_C−), as well as 8 active cell clones (DP) (Fig. 1b). Vector integration sites were sequenced for all cell clones, and cell clones with identical vector integration sites were treated as biological replicates, resulting in 7 M_TS_C+, 13 M_TS_C−, and 6 DP independent cell clones.

**Figure 4:**
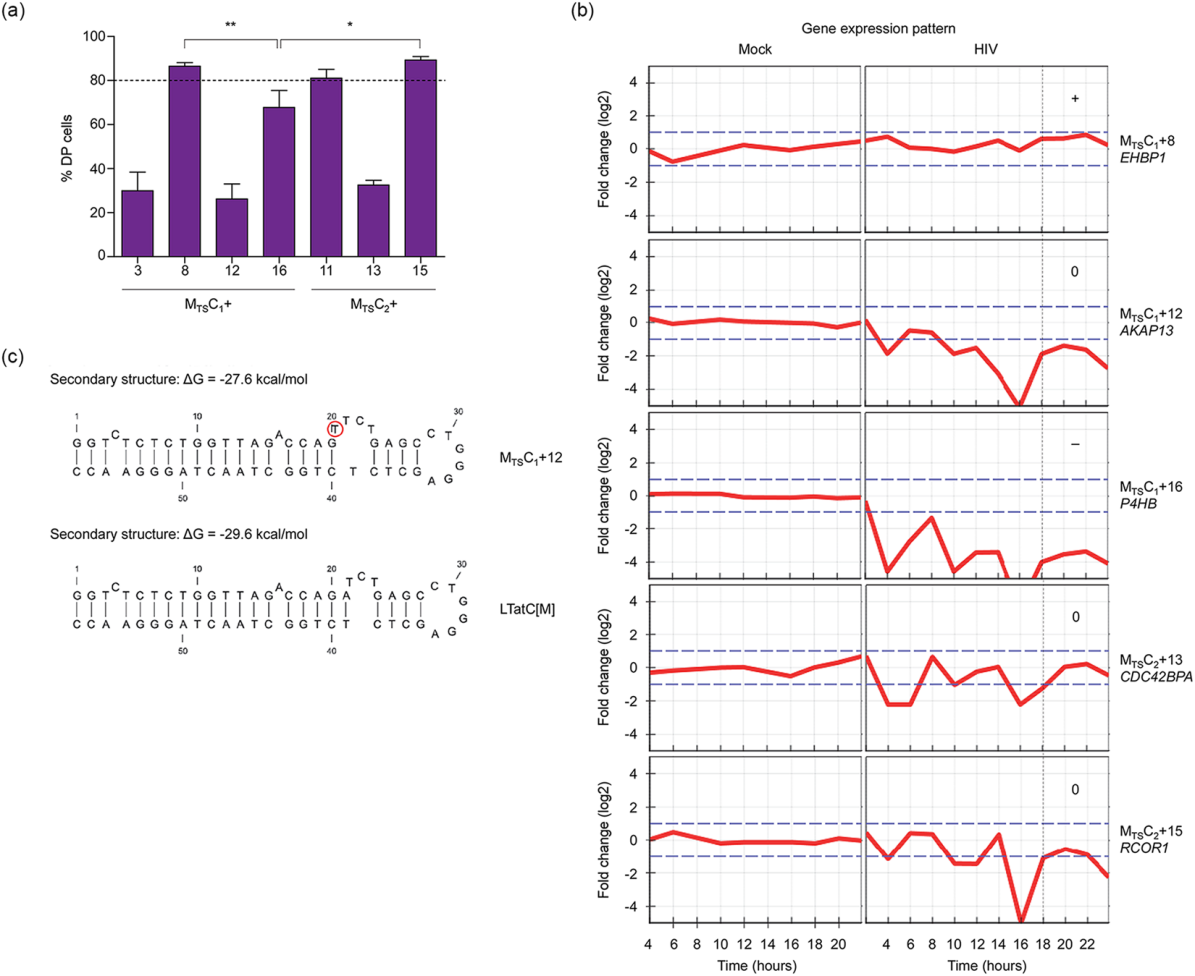
Factors influencing reactivation potentials of latent HIV-1 promoters in TNF-α and SAHA-responsive single mCherry positive (M_TS_C+) cell clones. (**a**) Reactivation potentials of latent HIV-1 promoters in TNF-α and SAHA-responsive single mCherry positive (M_TS_C+) cell clones with different LTatC[M] integration sites were reactivated with TNF-α and SAHA 54–106 days post sorting. The means of at least two independent measurements (n = 2–22) are indicated by the heights of the bars and standard error means are indicated by the error bars. Two-tailed Mann-Whitney *U* test with 95% confidence level was used to test for statistical significance; *P < 0.05; **P < 0.01. Subscripts 1 and 2 indicate two independent transduction and sorting experiments from which the cell clones were derived. DP: double positive. (**b**) Gene expression patterns in SUP-T1 cells during the first 24 hours upon infection with an HIV-1-based vector. Median log_2_ fold changes in expression of genes into which LTatC[M] was found integrated in different M_TS_C+ cell clones are shown. The majority of the HIV-1 integration process are defined as complete at 18 hours post infection. + and − indicate significant (*P* < 0.01) upregulation and downregulation in gene expression, respectively, whereas 0 indicates no significant changes in gene expression compared to data at time point 0 hour. Data were obtained from the Patterns of Expression and Analysis of Clusters of HIV/Host interactions (PEACHi) database^27^. Subscripts 1 and 2 indicate two independent transduction and sorting experiments from which the cell clones were derived. (**c**) Predicted RNA secondary structure of HIV-1 transactivation response element. The RNA secondary structures of HIV-1 transactivation response (TAR) element found in cell clone M_TS_C_1_+12 (top) and in the original vector LTatC[M] (bottom) were predicted using CLC Main Workbench 7. Red circle indicates the mutation in the TAR element of M_TS_C_1_+12.

We subsequently evaluated the reactivation potentials of latent HIV-1 promoters after expansion of M_TS_C+ and M_TS_C− cell clones in culture without TNF-α and SAHA, during which M_TS_C+ cell clones reverted to their initial phenotype of single mCherry positive. Surprisingly, highly variable reactivation potentials (26–89%) were observed among the seven M_TS_C+ cell clones with distinct vector integration sites (Fig. 4a; Table 1). To examine the effects of vector-hosting genes’ activity upon HIV-1 infection on the reactivation potentials of latent HIV-1 promoters, we used data from the Patterns of Expression and Analysis of Clusters of HIV/Host interactions (PEACHi) database^27^ (Fig. 4b). Data for M_TS_C_1_+3 (intergenic) and M_TS_C_2_+11 (*CTNND1/TMX2-CTNND1*) were not available on PEACHi. Of the five gene expression patterns for which data were available, *EHBP1* (M_TS_C_1_+8), *AKAP13* (M_TS_C_1_+12), *P4HB* (M_TS_C_1_+16), *CDC42BPA* (M_TS_C_2_+13), and *RCOR1* (M_TS_C_2_+15), only *P4HB* was significantly downregulated (~8-fold; *P*<0.01) post HIV-1 integration, *i.e*. > 18 hours post infection^27^ (Fig. 4b). While this could explain why M_TS_C_1_+16 had a significantly lower reactivation potential than M_TS_C_1_+8 and M_TS_C_1_+15, it did not explain the much lower reactivation potentials of M_TS_C_1_+12 and M_TS_C_2_+13 since the vector-hosting genes in these cell clones were not significantly downregulated post HIV-1 integration (Fig. 4a,b).

**Table 1:** Integration sites of LTatC[M] in TNF-α and SAHA-responsive single mCherry positive (M_TS_C+) cell clones.

Cell clone	Integration locus	Gene	Intragenic region	Transcriptional orientation
M_TS_C_1_+3	Chr3: 48710573	Intergenic	N/A	N/A
M_TS_C_1_+8	Chr2: 63216606	*EHBP1*	Intron 15/17*	Convergent
M_TS_C_1_+12	Chr15: 86127496	*AKAP13*	Intron 7	Same
M_TS_C_1_+16	Chr17:79808805	*P4HB*	Intron 4	Convergent
M_TS_C_2_+11	Chr11: 57536158	CTNND1/TMX2-CTNND1**	Intron 1/2**	Same
M_TS_C_2_+13	Chr1: 227375035	*CDC42BPA*	Intron 5	Same
M_TS_C_2_+15	Chr14: 103178189	*RCOR1*	Intron 7	Same

Two other cell clones, M_TS_C_1_+28 and M_TS_C_1_+34, and another cell clone, M_TS_C_2_+20, which were included in subsequent analyses, had the same LTatC[M] integration site as M_TS_C_1_+8 and M_TS_C_2_+11, respectively. *Transcript variant; **TMX2-CTNND1 is read-through long non-coding RNA. Subscripts 1 and 2 indicate two independent transduction and sorting experiments from which the clones were derived.

**Figure 5:**
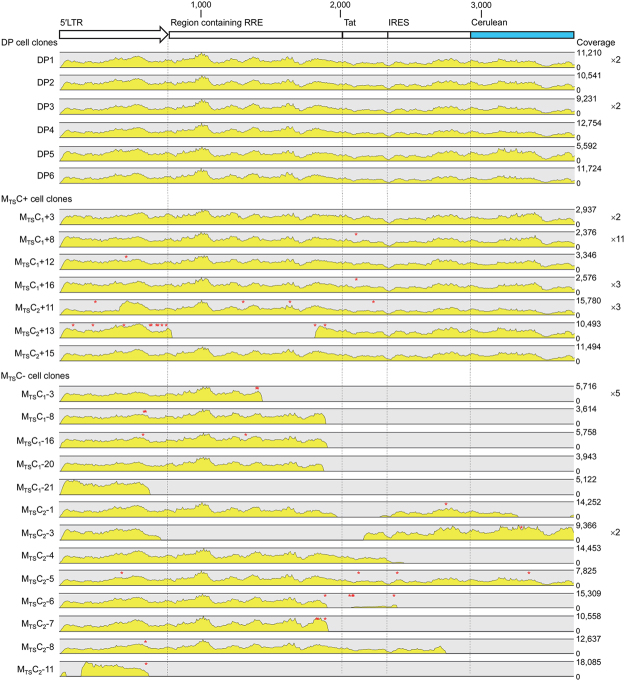
Mutational analysis of Cerulean cassettes of double positive (DP), TNF-α and SAHA-responsive single mCherry positive (M_TS_C+), and TNF-α and SAHA-non-responsive single mCherry positive (M_TS_C−) cell clones. Cerulean cassettes of double positive (DP), TNF-α and SAHA-responsive single mCherry positive (M_TS_C+), and TNF-α and SAHA-non-responsive single mCherry positive (M_TS_C−) cell clones were amplified and sequenced with the Illumina MiSeq next-generation sequencing technology. The schematic diagram of the LTatC[M] Cerulean cassette is shown on top and sequence coverages are depicted as yellow peaks with the range for each cell clone shown on the right. Point mutations are denoted by red asterisks. The numbers of cell clones with the same integration sites and mutation patterns analysed are shown next to the sequence coverage ranges. Subscripts 1 and 2 indicate two independent transduction and sorting experiments from which the cell clones were derived.

Therefore, we examined the Cerulean cassettes of all cell clones to determine whether mutations contributed to low reactivation potentials of latent HIV-1 promoters. None (0/6) of the DP cell clones analysed had any mutations in their Cerulean cassettes whereas mutations were found in 5/7 M_TS_C+ cell clones (Fig. 5). Notably, M_TS_C_1_+12 had a mutation in the HIV-1 transactivation response (TAR) element, which was predicted to disrupt the 3-nucleotide bulge essential for HIV-1 Tat binding and subsequent transcription elongation from the HIV-1 promoter^28,29^ (Fig. 4c), and M_TS_C_2_+13 had numerous mutations throughout its HIV-1 5′ LTR (Supplementary Table S1). Mutations in these cell clones could account for their low reactivation potentials. The mutations in the HIV-1 Tat region found in M_TS_C_1_+8 and M_TS_C_1_+16 (Fig. 5; Supplementary Table S1) have been reported to have wild-type transactivation activities^30,31^. Interestingly, no mutation was found in M_TS_C_1_+3 and M_TS_C_2_+15 while the reactivation potentials of latent HIV-1 promoters in these clones differed by ∼60% (Fig. 4a), further showing the influence of vector integration sites on the reactivation potentials of latent HIV-1 promoters. Taken together, our data provide evidence that the reactivation potentials of latent HIV-1 promoters are influenced by both vector integration sites and integrity of the Cerulean cassettes.

We then examined the Cerulean cassettes of the 18 M_TS_C− cell clones, the expression of which was not inducible with TNF-α and SAHA. The Cerulean cassettes of 17/18 cell clones contained large internal deletions in the HIV-1 Tat and/or Cerulean region (Fig. 5). Although cell clone MT_2_-5 did not have internal deletions, multiple mutations were found throughout its Cerulean cassette (Fig. 5), *e.g*. one in the stem of HIV-1 TAR and another resulting in a C37Y amino acid substitution in HIV-1 Tat, which likely abolished transactivation by Tat^28,29,31^. Thus, defective vector genome is the main cause of non-inducible HIV-1 promoters in M_TS_C− cell clones. This phenomenon has also been observed in cells from HIV-1-infected individuals^4^.

### Higher expression of a distinct set of cell cycle regulators in TNF-α and SAHA-responsive single mCherry positive (M_TS_C+) cell subpopulations with spontaneously reactivated HIV-1 promoters

**Figure 6:**
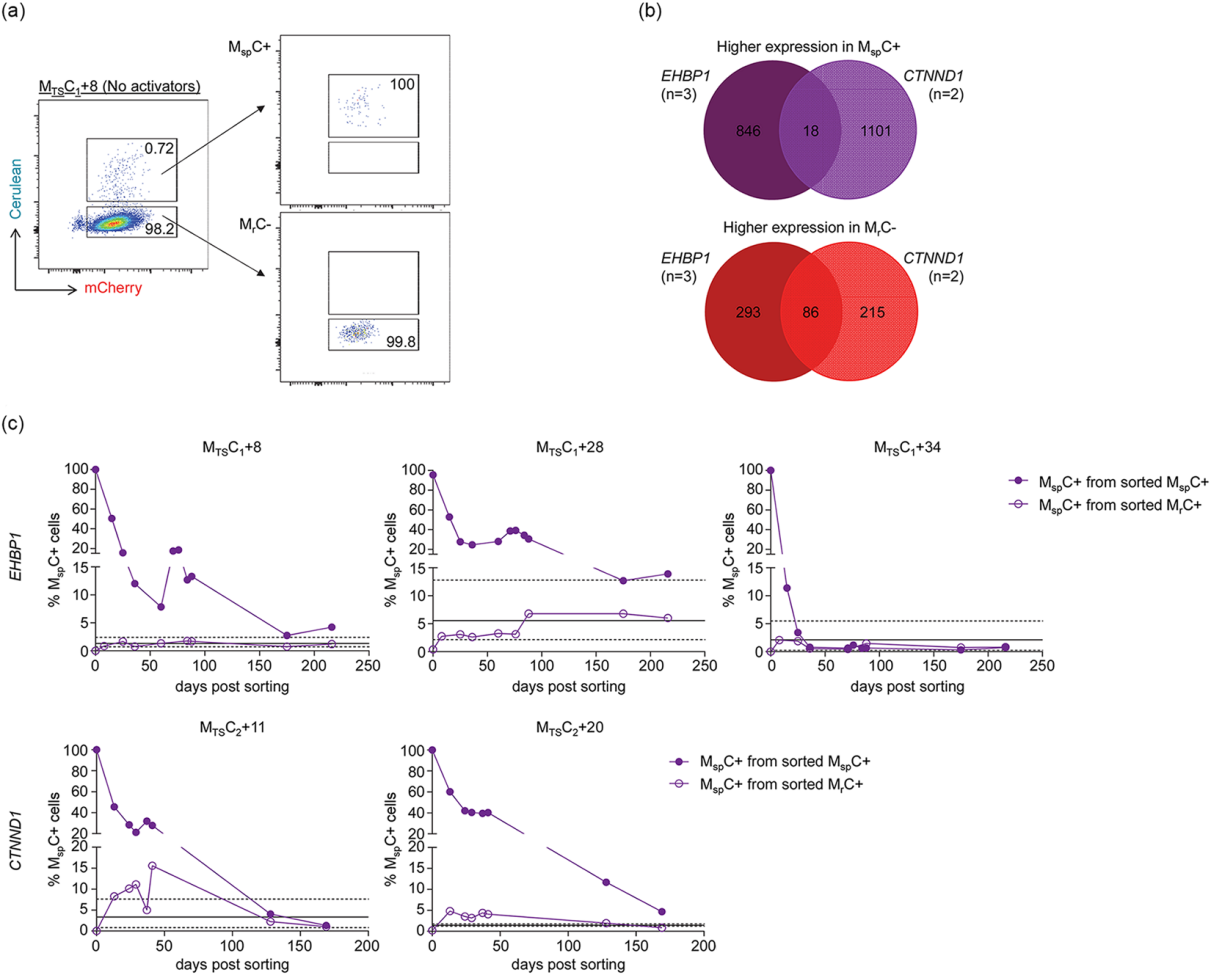
Longitudinal fluorescence profiles of spontaneous double positive (M_sp_C+) cell subpopulations and their counterparts that remained single mCherry positive (M_r_C−) sorted from TNF-α and SAHA-responsive single mCherry positive (M_TS_C+) cell clones. TNF-α and SAHA-responsive single mCherry positive (M_TS_C+) cell clones that exhibited a small subpopulation of spontaneous double positive (M_sp_C+) cells without treatment with activators were sorted by flow cytometry for M_sp_C+ cells and single mCherry cells that did not spontaneously become DP, *i.e*. non-spontaneous M+ (M_r_C−). (**a**) Representative flow cytometric sorting strategy and purity of M_sp_C+ and M_r_C− cell subpopulations from M_TS_C_1_+8 cell clone. (**b**) Number of genes that had a higher expression level in M_sp_C+ (top) and M_r_C− (bottom) cell subpopulations and were common between three M_TS_C+ cell clones with LTatC[M] integrated into *EHBP1* and two M_TS_C+ cell clones with LTatC[M] integrated into *CTNND1*. (**c**) Longitudinal percentages of M_sp_C+ cell subpopulations remaining and emerging from sorted M_sp_C+ (shaded circles) and M_r_C− (open circles), respectively. Solid and dashed horizontal lines indicate the means and ranges of at least five independent measurements (n ≥ 5) of percentages of M_sp_C+ cells in the unsorted parental cell clones. The top three cell clones had the same LTatC[M] integration site in *EHBP1* whereas the bottom two cell clones had the same LTatC[M] integration site in *CTNND1*. Subscripts 1 and 2 indicate two independent transduction and sorting experiments from which the cell clones were derived.

Some M_TS_C+ cell clones when reverted to their initial M+ phenotype upon TNF-α and SAHA withdrawal, constantly exhibited a small subpopulation of cells with a spontaneously reactivated HIV-1 promoter, *i.e*., cells that became double positive without further external stimulation. These spontaneous double positive cells are termed M_sp_C+ while the majority of cells that remained single mCherry positive are termed M_r_C− here (Fig. 6a). To examine whether M_sp_C+ and M_r_C− phenotypes were reversible, we sorted out the two cell subpopulations respectively from five M_TS_C+ cell clones representing two distinct vector integration sites: M_TS_C_1_+8, M_TS_C_1_+28, and M_TS_C_1_+34 which had LTatC[M] integrated into *EHBP1*, and M_TS_C_2_+11 and M_TS_C_2_+20 which had LTatC[M] integrated into *CTNND1* (Fig. 6a; Table 1). The percentages of spontaneous double positive cells that remained in the sorted M_sp_C+ cell subpopulations and those that emerged from the sorted M_r_C− cell subpopulations over time were monitored with flow cytometry. For all M_TS_C+ cell clones, the majority of the sorted M_sp_C+ cells became single mCherry positive while a small fraction of M_r_C− cells became double positive to the extents of their respective parental cell clones (Fig. 6c).

Given that the M_sp_C+ and M_r_C− phenotypes were reversible, we hypothesized that the spontaneous reactivation of HIV-1 promoters was linked to the cell cycle. The transcriptomes of M_sp_C+ and M_r_C− cells at 0 day post sorting were sequenced to identify differentially expressed genes between the two cell subpopulations (Supplementary Fig. S3). Cells of the same fluorescence phenotype and vector integration site were treated as biological replicates. Using the cut-offs of fold change >1.5× (|log_2_ratio| > 0.585) and *P* < 0.02, we identified 18 genes that had a higher expression level in M_sp_C+ cell subpopulations compared to M_r_C− and were common between the two vector integration sites (Fig. 6b; Supplementary Table S2a). Of these 18 genes, three (16.7%) are known to promote cell cycle progression: *FOSB*, *NEAT1*, and *EGR1* (Supplementary Table S2a). For instance, the two top hits, *FOSB* and *NEAT1*, have been reported to induce cell cycle entry by activating cyclin D1 in mouse fibroblasts^32^ and promote cell proliferation in cancer cells^33^, respectively. Furthermore, *NEAT1* and *EGR1* have also been reported to be involved in the HIV-1 replication cycle^34,35^. On the other hand, 86 genes were found to have a higher expression level in M_r_C− cell subpopulations (Fig. 6b; Supplementary Table S2b). Of these 86 genes, 81 encode non-coding RNAs (Supplementary Table S2b), such as *RN7SK*, the RNA product of which sequesters CDK9 and CycT1 from acting as positive transcription elongation factors at the HIV-1 promoter^36^, and *RN7SL1*, *RN7SL2*, *RNY1*, and *RNY3*, which are cellular RNAs typically co-packaged in the HIV-1 viral particle^37^. Six (7.0%) and three (3.5%) of these 86 genes are known to promote and suppress cell cycle progression, respectively (Supplementary Table S2b).

### Larger fractions of spontaneous double positive (M_sp_C+) cells are in the S and G2 phases of the cell cycle compared to cells remaining as single mCherry positive (M_r_C−) and treatment with cell cycle regulators increased spontaneous reactivation

**Figure 7:**
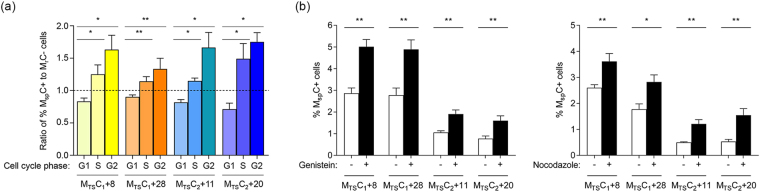
Spontaneous reactivation of latent HIV-1 promoters is linked to the cell cycle. (**a**) Ratio of percentages of cells in the various phases of the cell cycle in spontaneous double positive (M_sp_C+) to non-spontaneous M+ (M_r_C−) cell subpopulations. The means of at least four independent measurements (n ≥ 4) are indicated by the heights of the bars and standard error means are indicated by the error bars. (**b**) Percentages of M_sp_C+ cells upon treatment with genistein (left) and nocodazole (right) in comparison to equally diluted DMSO control (−). The means of five independent measurements (n = 5) are indicated by the heights of the bars and standard error means are indicated by the error bars. Two-tailed Mann-Whitney *U* test with 95% confidence level was used to test for statistical significance; *P < 0.05; **P < 0.01. Subscripts 1 and 2 indicate two independent transduction and sorting experiments from which the cell clones were derived. M_TS_C+: TNF-α and SAHA-responsive single mCherry positive.

To further explore the linkage between spontaneous activation of latent HIV-1 promoters and the cell cycle, four M_TS_C+ cell clones representing two vector integration sites were resolved into G1, S, and G2 phases of the cell cycle by flow cytometric analysis of cells with their genomic DNA content fluorescently labelled. A significant increase in percentages of cells in the S (*P* < 0.05) and G2 (*P* < 0.001) phases of the cell cycle was observed in M_sp_C+ cells in comparison to M_r_C− cells (Supplementary Fig. S4). The ratio of percentages of M_sp_C+ cells to M_r_C− cells in each of the three cell cycle phases are shown in Fig. 7a. Consistently, treatment of these cell clones with genistein and nocodazole, which halted SUP-T1 cells in the G2 phase of the cell cycle (Supplementary Fig. S5), increased the percentages of spontaneous reactivation by 1.8–2.1- and 1.4–2.9-fold, respectively (Fig. 7b). Taken together with the transcriptomic data above, our findings suggest that spontaneous activation of latent HIV-1 promoters is linked to the cell cycle.

## Discussion

HIV-1-based vectors have previously been utilized to dissect various aspects of HIV-1 infection^7,10,11^. In the present study, we have developed a novel vector, LTatC[M], and characterized it in great detail with regard to its representation of HIV-1 infection and latency in SUP-T1 cells. In contrast to previous vectors, LTatC[M] contains a pair of genetic insulators, cHS4 and sMAR, that flank the constitutive mCherry cassette to protect it from position-effect variegation that might be exerted at some integration sites^7,38^. Additionally, mCherry expression is driven by the human *eIF4A1* promoter, which has been shown to yield high levels of gene expression in macrophage cell lines^39^, potentially enabling the use of LTatC[M] to study HIV-1 infection in this other target cell type of HIV-1. Furthermore, cHS4 is capable of alleviating transcriptional interference between tandem gene cassettes^17–19^. The functionality of the genetic insulators was evident in two ways: (i) stable expression of Cerulean and/or mCherry in long-term bulk and monoclonal cell cultures for up to 394 days and (ii) absence of reactivatable HIV-1 and *heIF4A1* promoters in the double negative cell population. In contrast, in a previous study using a dual-fluorescence vector without genetic insulators, a substantial fraction of double negative cells was found to harbour a reactivatable vector^40^. More importantly, these genetic insulators did not alter the kinetics and ratio of emergence of active and latent cells and vector integration site patterns compared to the control vector lacking the genetic insulators. With our model, we were able to examine HIV-1 latency in monoclonal cells with distinct vector integration sites. We focused on factors that contribute towards two aspects of HIV-1 latency reversal: (i) spontaneous reactivation and (ii) reactivation potentials upon external induction.

Spontaneous reactivation of latent HIV-1 promoters *in vitro* has also been observed by others^7^, although a mechanistic explanation is lacking. We found that spontaneous reactivation of latent HIV-1 promoters is linked to the cell cycle, as evidenced by transcriptomic profiling to identify differentially expressed genes between spontaneously reactivated and the remaining non-activated cell subpopulations within cell clones. This linkage was further corroborated by two observations: (i) larger fractions of spontaneously reactivated cells were in the S and G2 phases of the cell cycle compared to the non-activated cells and (ii) halting the cell cycle at the G2 phase with genistein and nocodazole increased the fraction of spontaneous reactivation. This is analogous to the partial reactivation of latent HIV-1 observed in central memory CD4^+^ T cells undergoing homeostatic proliferation^41^. Furthermore, viral production is known to increase in the G2 phase^42,43^. Interestingly, two cell cycle regulators having a higher expression level in spontaneously reactivated cells, *NEAT1* and *EGR1*, have also been reported to be involved in the HIV-1 replication cycle. *NEAT1* is essential for the formation of nuclear paraspeckles and has been found to be upregulated in peripheral blood mononuclear cells of untreated HIV-1-infected individuals (productive infection) compared to those of virally suppressed (latent infection)^44^. Additionally, knockdown of *NEAT1* enhances HIV-1 production^35^. These studies suggest that *NEAT1* might be upregulated as an antiviral response to HIV-1 expression, *e.g*. HIV-1 Tat in spontaneously reactivated cells. *EGR1* encodes a transcriptional factor that regulates genes related to cell growth and differentiation. The expression of *EGR1* is upregulated by HIV-1 Tat^45,46^ and its activation leads to the reversal of HIV-1 latency^34^, consistent with our observation that *EGR1* was expressed more in spontaneously reactivated cells. The vast majority of genes identified to have a higher expression level in non-activated cells were non-coding small nuclear RNAs (snRNAs) with unknown relevance to HIV-1 infection, except for *RN7SK*, which forms an RNA scaffold that binds CDK9 and CycT1, thus sequestering these molecules from acting as a positive transcription elongation factor at the HIV-1 promoter^36^. The role of non-coding RNAs, especially long non-coding RNAs and microRNAs, in the modulation of HIV-1 infection is of great interest^47,48^ and snRNAs identified in our screen might represent an additional layer of potential candidates for further investigation in the context of HIV-1 latency establishment and maintenance.

The observations that larger fractions of spontaneously reactivated cells are in the later phases of the cell cycle and that spontaneous reactivation increased upon treatment with cell cycle regulators suggest that cell cycling might lead to spontaneous reactivation. This would provide a mechanistic explanation to the spontaneous reactivation of latent HIV-1 proviruses observed in HIV-1-infected individuals during treatment interruption as a result of homeostatic proliferation of latently infected cells. Our model, however, does not exclude the alternative; cell cycling is a result of spontaneous reactivation. Spontaneous reactivation of latent HIV-1 proviruses has been described as an intrinsic characteristic of the virus^49,50^. We detected a higher expression of *EGR1*, which is a positive cell cycle regulator, in spontaneously reactivated cells and upregulation of *EGR1* by HIV-1 Tat has been shown^45,46^. *Tat* is contained in our vector and it is an early-expressed gene in the HIV-1 replication cycle^36^. Thus, the intrinsic spontaneous reactivation of latent HIV-1 proviruses may drive cell cycling, potentially contributing towards clonal expansion of latently infected cells^51–53^. Of note, our minimalistic HIV-1-based vector does not encode, for example, HIV-1 *Vpr*, which is also known to modulate the host cell’s cell cycling programme^54^. Nonetheless, individual components of the viral genome could be cloned into our vector, thus enabling the possibility to systematically examine individual or combined effects of these components on cell cycling as well as other aspects of the host cell.

HIV-1 integration site selection has been implicated to have an influence on whether the integrated provirus would be active or latent, as well as the reactivation potentials of latent proviruses^13,14^. Vector integration sites identified in our model conformed to HIV-1 integration site patterns^8,26,55–57^. Furthermore, cells representing latent HIV-1 infections consistently exhibited different vector integration site patterns, albeit modest, than their active counterparts in two independent transductions; higher frequencies in transcription units and in the same transcriptional orientation relative to vector-hosting genes. These differences between active and latent cells were also reported in a primary-cell model transduced with HIV-1_NL4–3_ whose env was partially replaced with a fluorescent reporter gene^58^. More HIV-1 integrations in a convergent transcriptional orientation have also been observed in viraemic (active infections) than in treated (latent infections) individuals^55^. We further dissected cells with a latent HIV-1 phenotype into those that were inducible with TNF-α and SAHA to become active and those that were non-inducible. Inducible cell clones with distinct vector integration sites and expression profiles of vector-hosting genes displayed variable reactivation potentials. The reactivation potentials were dramatically attenuated in inducible cell clones with mutations in elements important for the expression of Cerulean, demonstrating that the integrity of the viral genome, in addition to features of the viral integration site, has an impact on HIV-1 latency reversal. On the other hand, we did not find any non-inducible cell clone with an intact Cerulean cassette, although the sample size of cells analysed was relatively small. Major internal deletions and mutations, possibly a result of the error-prone HIV-1 reverse transcriptase^59,60^ and copy-choice recombination^61,62^, were the cause of these clones not being reactivatable. Such a phenomenon is also observed in cells derived from HIV-1-infected individuals^4^, although the rate and site of mutation and recombination are likely to differ in our system utilizing a minimalistic HIV-1-based vector. Nonetheless, our observation accentuates the need to carefully examine singly fluorescent cell populations, *e.g*. latency-representing cells, in HIV-1 latency models using HIV-1-based vector systems.

Besnard *et al*. recently, in a genome-wide shRNA screen, identified candidate genes that either promote or inhibit HIV-1 latency, and subsequently showed that the mechanistic target of rapamycin (mTOR) complex is a positive regulator of HIV-1 latency reversal^63^. In their screen, actin cytoskeleton reorganization pathways were also found to be potentially involved in the regulation of HIV-1 latency^63^. Interestingly, 4/7 inducible cell clones analysed had LTatC[M] integrated into genes related to actin cytoskeleton reorganization: *EHBP1*^64^, *AKAP13*^65^, *CTNND1*^66^, and *CDC42BPA*^67^. For example, EHBP1 links actin to the cell membrane for endosomal tubulation^64^ and CDC42BPA (Cell division control 42 binding protein kinase α) (CDC42 effector protein 5 is the second highest latency-promoting gene in the study of Besnard *et al*.)^63^ promotes actin and myosin reorganization^67^. These four genes are also listed as latency promoting in the study of Besnard *et al*.^63^. The linkage between integration into these genes and HIV-1 latency establishment is currently unclear. Nevertheless, our observation complements current knowledge on HIV-1 latency and presents an interesting finding for further investigation.

## Conclusions

In summary, our well-characterized dual-fluorescence, genetic-insulators-containing HIV-1-based vector is capable of recapitulating various facets of HIV-1 infection and latency and invariably maintains active and latent HIV-1 phenotypes over a long observation period spanning at least one year. Application of this vector might provide insights into the pathomechanisms of HIV-1 latency. We applied this vector to study spontaneous reactivation of latent HIV-1 promoters in SUP-T1 cells and found a linkage between this phenomenon and the cell cycle. This is an important basis for further investigations on the mechanisms of maintenance of the HIV-1 reservoir and by which spontaneous viral rebound occurs in HIV-1-infected individuals off ART.

## Methods

### Plasmid vector construction

LTatC[M] was synthesized by GeneArt (Thermo Fisher Scientific), except that the HIV-1 Rev response element (RRE) was amplified and cloned from pEV731^7^ (kindly provided by Eric Verdin) with primer pair 5′NNNNNNGTCGACCTCGAGATGGGTGCGAGAGCGTCAG3′ and 5′NNNNNNGTCGACGGTGGCATCGATACCGTCGAG3′, and the Woodchuck hepatitis virus post-transcriptional regulatory element (WPRE) was amplified from pCLX-UBI-GFP (kindly provided by Patrick Salmon; Addgene plasmid # 27245) with primer pair 5′NNNNNNGGATCCCGATAATCAACCTCTGGATTAC3′ and 5′NNNNNNACCGGTAATTCCCAGGCGGGGAGG3′. Genetic elements in LTatC[M] are shown in Fig. 1a and tabulated in Supplementary Table S3.

### Virus pseudotyping and titration

VSV-G-pseudotyped LTatC[M] were generated by transfecting 293 T cells (American Type Culture Collection) of at least 90% confluency cultured in DMEM (Sigma-Aldrich) supplemented with 10% (v/v) fetal bovine serum (FBS) (Thermo Fisher Scientific), 100 U/mL Penicillin, and 100 μg/mL Streptomycin (Thermo Fisher Scientific) in a T-150 flask with the following components: pLTatC[M] (20.8 μg), psPAX2 (20.8 μg) (kindly provided by Didier Trono; Addgene plasmid # 12260), pCAG-VSVG (10.4 μg) (kindly provided by Arthur Nienhuis and Patrick Salmon; Addgene plasmid # 35616), polyethylenimine (208 μg), and serum-free DMEM in a total volume of 3.4 mL. Medium was replaced 18 hours post transfection. Supernatant containing viral particles was harvested twice at 24 and 48 hours post medium change, and concentrated with PEG-it (System Biosciences) to a final volume of 450 μL.

VSV-G-pseudotyped virus stocks were titrated by transducing 0.2 million SUP-T1 cells^68^ (kindly provided by Dharam Ablashi through the NIH AIDS Reagent Programme, Division of AIDS, NIAID, NIH) cultured in 200 μL of RPMI-1640 (Sigma-Aldrich) supplemented with 10% (v/v) FBS, 100 U/mL Penicillin, and 100 μg/mL Streptomycin in 48-well plates with virus stocks. Medium was replaced daily and cells were analysed with flow cytometry (BD Biosciences LSR Fortessa) for total positive cells, *i.e*., cells expressing mCherry and/or Cerulean, at 5 days post transduction. The mean viral titre was calculated from transductions that yielded a total positive cells of <20%, thus ensuring a high probability of obtaining single integrants per cell^69^, and expressed as SUP-T1 transduction units/mL.

### SUP-T1 transduction and flow cytometric sorting

SUP-T1 cells were transduced with VSV-pseudotyped LTatC[M] to yield 5–56% of positive cells. All four cell populations were sorted with flow cytometry (BD Biosciences FACS ARIA III) 4–13 days post transduction. For sorting of tumour necrosis factor alpha (TNF-α) (Bio-Techne) and suberanilohydroxamic acid (SAHA) (Bio-Techne)-inducible (M_TS_C+) and -non-inducible (M_TS_C−) cell populations, M+ cells were treated with 0.01 μg/mL TNFα and 1 μM SAHA for 24 hours prior to sorting.

### Amplification, sequencing, and mapping of 5′ vector integration junctions

The 5′ vector integration junctions were amplified, sequenced, and mapped as described previously^26^. Briefly, 5′ vector integration junctions were amplified with the non-restrictive linear amplification-mediated PCR^70^ and 12–14 pM purified PCR products were sequenced with the Illumina MiSeq platform using the MiSeq Reagent Kit v2 (300 cycles) (Illumina) with 8% PhiX. Mapping of sequencing reads to the human genome assembly GRCh37.p13 was performed using an in-house bioinformatic pipeline, Integration Site Analysis Pipeline (InStAP). All vector integration junctions are listed in Supplementary Table S4.

### Amplification and sequencing of Cerulean cassettes

The Cerulean cassettes, spanning from the HIV-1 5′ LTR to cHS4, in transduced cells were amplified using Platinum Taq DNA polymerase high fidelity (Thermo Fisher Scientific) with the forward primer 5′GACAAGAGATCCTTGATCTGTGGATC3′ and reverse primer 5′CACTGATAGGGAGTAAACATATGC3′ or 5′GAAGGACAGCTTCAAGTAGTCG3′. The cycling conditions were 94 °C for 2 min, 40 cycles of 94 °C for 30 s, 55 °C for 30 s and 68 °C for 5 min 30 s, and a final extension of 68 °C for 10 min.

The amplicons were processed with the Nextera XT DNA Library Preparation Kit (Illumina) and subsequently sequenced with the MiSeq reagent Kit v2 (50 cycles) with 1% PhiX.

### Transcriptomic analysis

Total RNA was extracted using AllPrep DNA/RNA Kit (Qiagen) and reverse-transcribed library was prepared and normalized using SMARTer Stranded Total RNA-Seq Kit – Pico Input Mammalian (Takara). The TruSeq SR Cluster Kit v4-cBot-HS or TruSeq PE Cluster Kit v4-cBot-HS (Illumina) was used for cluster generation with 8 pM pooled normalized libraries on the Illumina cBOT system. Sequencing was performed with the Illumina HiSeq. 2500; paired end at 2 × 126 bp or single end 126 bp using the TruSeq SBS Kit v4-HS (Illumina).

Bioinformatic analysis was performed using the R package ezRun (https://github.com/uzh/ezRun) within the data analysis framework SUSHI^71^. Raw reads were quality checked using Fastqc (http://www.bioinformatics.babraham.ac.uk/projects/fastqc/) and FastQ Screen (http://www.bioinformatics.babraham.ac.uk/projects/fastq_screen/). Quality-controlled reads were aligned to the reference genome GRCh38 using the STAR aligner^72^. Expression counts were computed using featureCounts in the Bioconductor package Subread^73^. Differential expression analysis was performed using the edgeR package^74^, where raw read counts were normalized using the Trimmed Mean of M values (TMM) method^75^ and differential expression was computed using the Generalized Linear Mode (GLM) likelihood ratio test. Quality checkpoints^76^, such as quality control of the alignment and count results were implemented in ezRun and applied throughout the analysis workflow to ensure correct data interpretation.

### Cell cycle analysis

A total of 1 million cells were fixed in 1 mL of 2% (w/v) paraformaldehyde (Fluka) in Dulbecco’s phosphate-buffered saline (DPBS) on ice for 15 min, washed with DPBS twice by centrifugation at 800 g for 5 min, resuspended in 1 mL of 0.1% (v/v) Triton X-100 (Sigma-Aldrich) in DPBS supplemented with 1% (v/v) FBS, treated with 0.1 mg/mL RNase A (Thermo Fisher Scientific), and stained with 1 μL of FxCycle Far Red Stain (Thermo Fisher Scientific) at room temperature for 30 min. Cell cycle profiles were modeled with the Cell Cycle function in FlowJo v10.0.8 [Watson (Pragmatic); CV of G2 peak = CV of G1 peak].

### Cell cycle regulators treatment

A total of 0.1 million M_TS_C+ cells cultured in 200 μL of RPMI-1640 (10% (v/v) FBS, 100 U/mL Penicillin, and 100 μg/mL Streptomycin) or 1 million SUP-T1 cells cultured in 2 mL of RPMI-1640 (10% (v/v) FBS, 100 U/mL Penicillin, and 100 μg/mL Streptomycin) were treated with 30 μM genistein (Selleckchem), 40 ng/mL nocodazole (Sigma-Aldrich), or equally diluted dimethyl sulfoxide (DMSO) (Sigma-Aldrich) for 24 hours prior to flow cytometric analysis.

### Data availability

Data generated and analysed during this study are included in this published article and its supplementary information files unless stated otherwise, in which case are available from the corresponding author on reasonable request.

## Electronic supplementary material

Supplementary Information

Supplementary Table S4
