# Supplementary material for: Spontaneous reactivation of latent HIV-1 promoters is linked to the cell cycle as revealed by a genetic-insulators-containing dual-fluorescence HIV-1-based vector
Source: Sci Rep. 2018 Jul 5;8:10204. doi: 10.1038/s41598-018-28161-y (PMC6033903; doi:10.1038/s41598-018-28161-y)
Supplement: Supplementary file 1 — Supplementary Information [file 41598_2018_28161_MOESM1_ESM.docx]

**Spontaneous reactivation of latent HIV-1 promoters is linked to the cell cycle as revealed by a genetic-insulators-containing dual-fluorescence HIV-1-based vector**

**Supplementary Information**

Yik Lim Kok^1,2^, Stefan Schmutz^2^, Anne Inderbitzin^1,2,3^, Kathrin Neumann^1,2^, Audrey Kelley^1,2,3^, Lisa Jörimann^1,2^, Mohaned Shilaih^1,2^, Valentina Vongrad^1,2^, Roger D. Kouyos^1,2^, Huldrych F. Günthard^1,2^, Christian Berens^4^ & Karin J. Metzner^1,2*^

**Affiliations**

^1^Division of Infectious Diseases and Hospital Epidemiology, University Hospital Zurich, Zurich, Switzerland.

^2^Institute of Medical Virology, University of Zurich, Zurich, Switzerland.

^3^Life Science Zurich Graduate School, University of Zurich, Zurich, Switzerland.

^4^Institute of Molecular Pathogenesis, Friedrich-Loeffler-Institut, Jena, Germany.

*Address correspondence to Karin J. Metzner, [karin.metzner@usz.ch](mailto:karin.metzner@usz.ch).

**
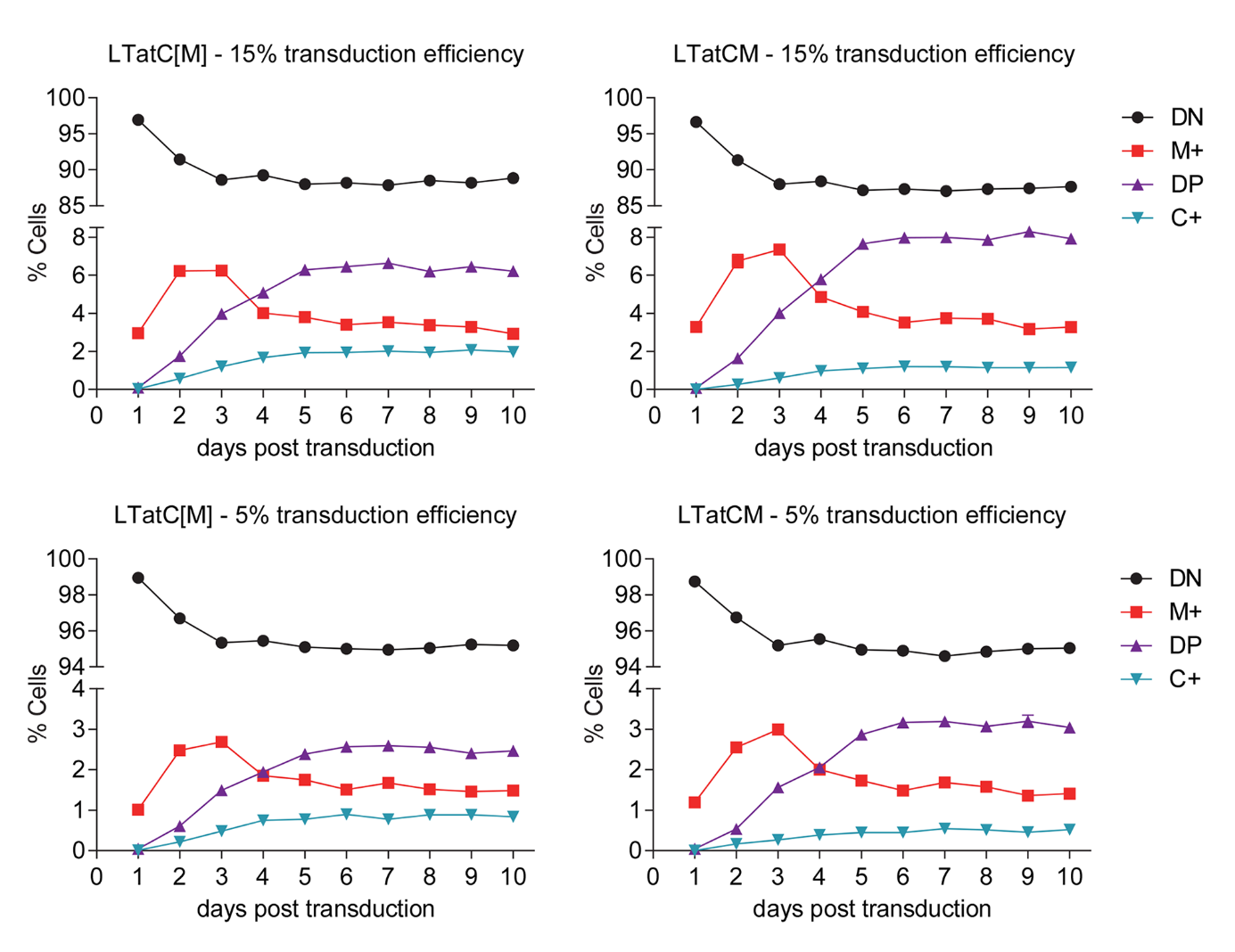
Supplementary Fig. S1: 10-day fluorescence profile of SUP-T1 cells transduced with LTatC[M] or LTatCM.**

SUP-T1 cells were transduced with LTatC[M] (left panel) or a vector variant with no genetic insulators in the mCherry cassette (LTatCM) (right panel) at 15% (top panel) or 5% (bottom panel) transduction efficiencies. The fluorescence profiles of the four cell populations arose from each transduction: double negative (DN), single mCherry positive (M+), double positive (DP), and single Cerulean positive (C+), were measured with flow cytometry daily for 10 days. Each datapoint represents the mean of two independent transductions (n=2) and error bars depict standard error means. Some error bars are within datapoints. The upper left graph is also shown as Fig 1c.

**
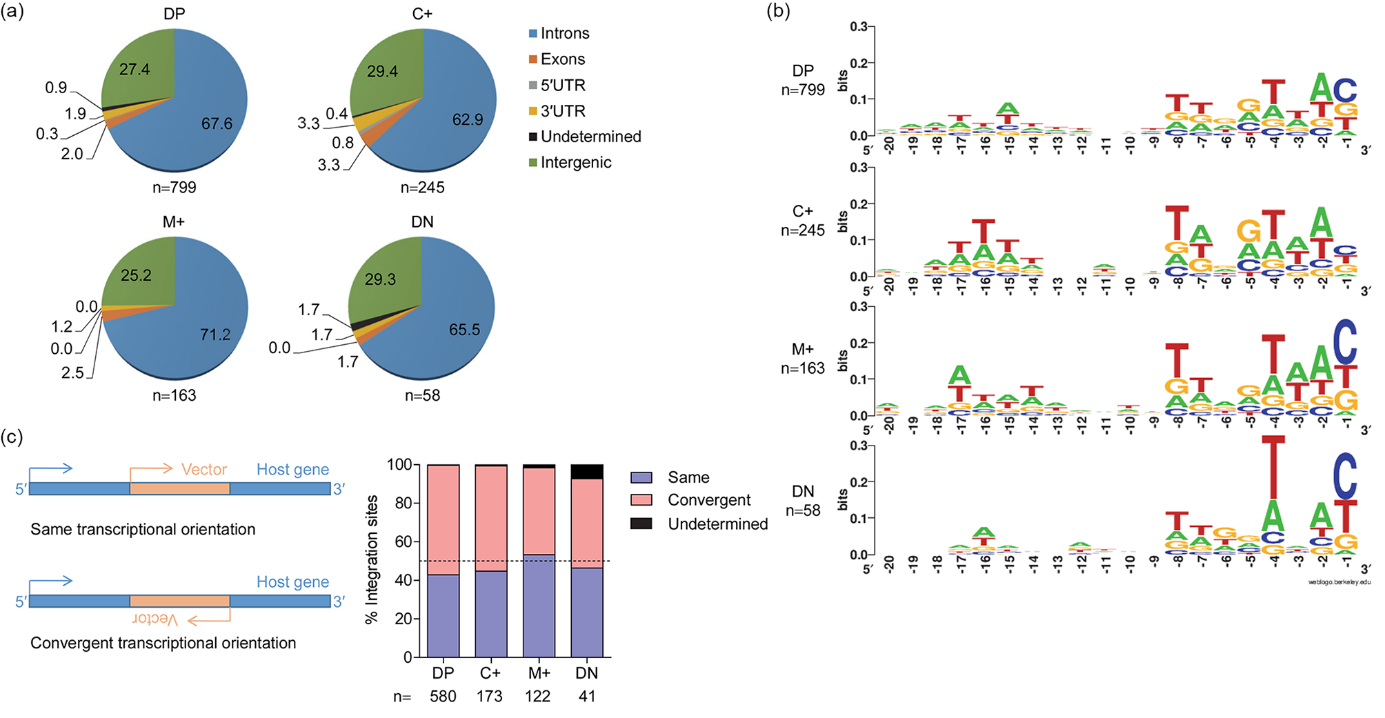
**

**Supplementary Fig. S2: Integration site patterns of LTatC[M] in various SUP-T1 cell populations in a second transduction experiment.**

(A) Genomic distributions of integrated LTatC[M]. (B) Consensus sequences of 20 nucleotides upstream of 5′LTR. (C) Transcription orientations of intragenic LTatC[M] relative to its host genes. DP: double positive; C+: single Cerulean positive; M+: single mCherry positive.

**
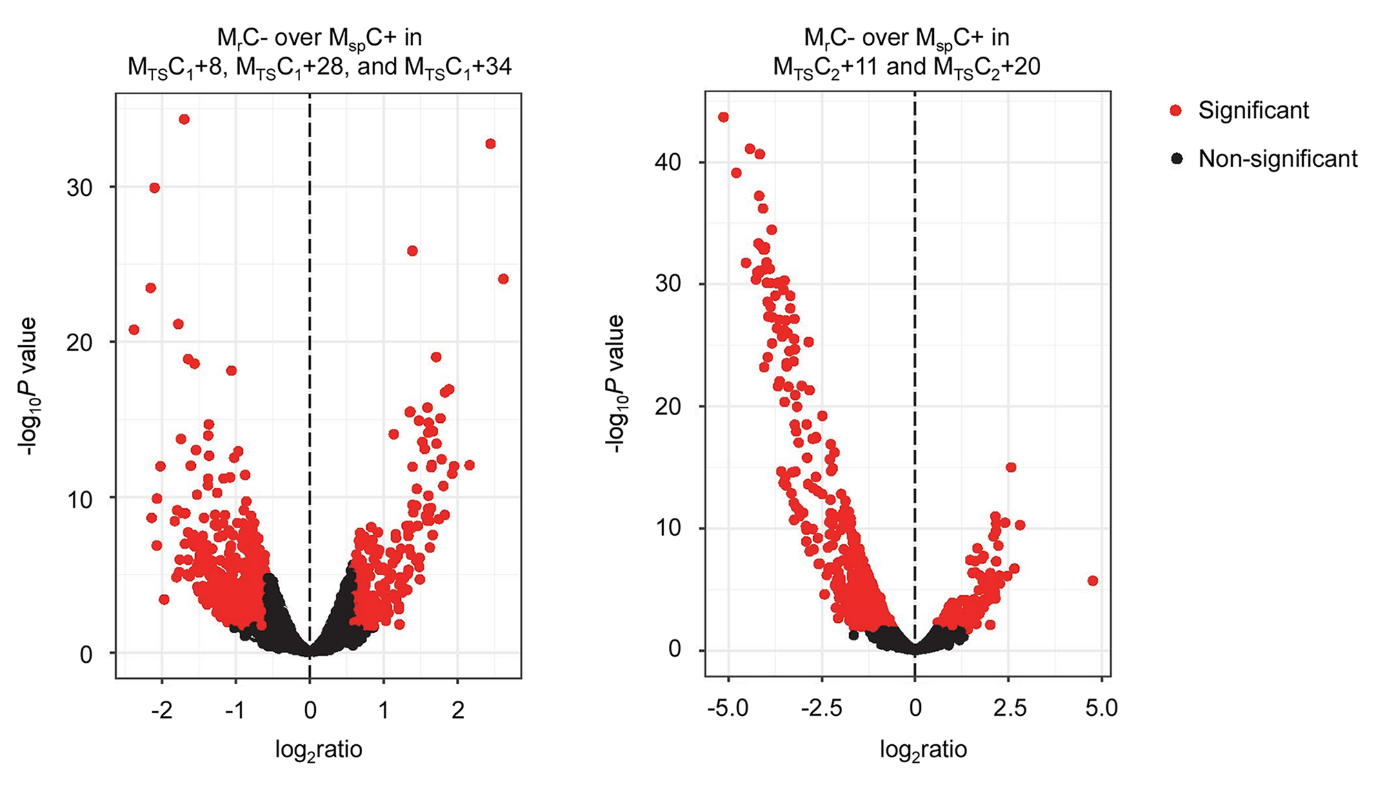
**

**Supplementary Fig. S3: Transcriptomic sequencing to identify differentially expressed genes between spontaneous double positive (M_sp_C+) cell subpopulations and their counterparts that remained single mCherry positive (M_r_C-).**

Volcano plots show genes that had a significantly (*P*<0.02; |log_2_ratio|>0.585) higher expression in non-spontaneous M+ (M_r_C-), *i.e.* single mCherry cells that did not spontaneously become double positive, (positive log_2_ratio) and spontaneous double positive (M_sp_C+) (negative log_2_ratio) cell subpopulations of TNF-α and SAHA-responsive single mCherry positive (M_TS_C+) cell clones. Each cell subpopulation with the same LTatC[M] integration site was treated as biological replicates. M_TS_C_1_+8, M_TS_C_1_+28, and M_TS_C_1_+34 had the same LTatC[M] integration site in *EHBP1* whereas M_TS_C_2_+11 and M_TS_C_2_+20 had the same LTatC[M] integration site in *CTNND1*. The generalized linear mode likelihood ratio test implemented in the EdgeR package was used to determine differentially expressed genes. Subscripts 1 and 2 indicate two independent transduction and sorting experiments from which the cell clones were derived.


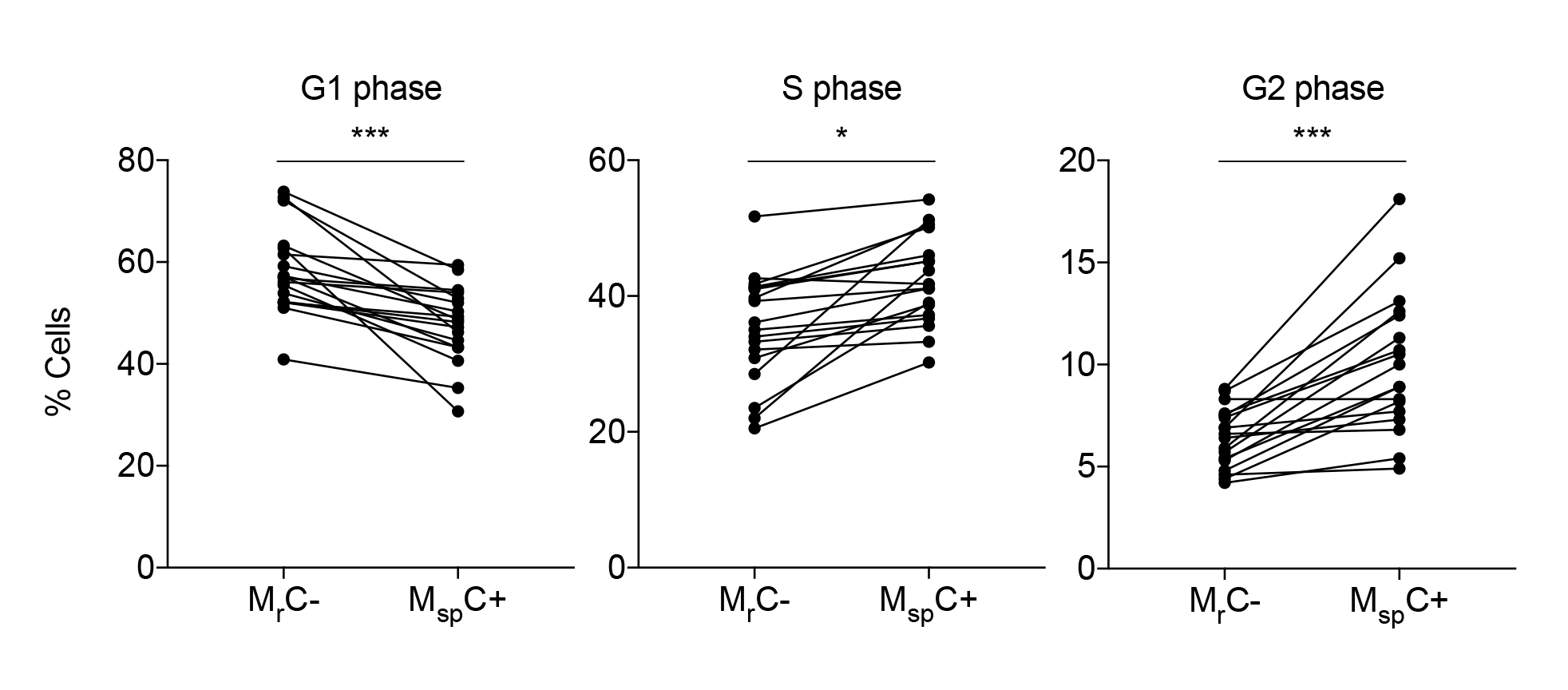


**Supplementary Fig. S4: Percentages of cells in the various phases of the cell cycle in non-spontaneous M+ (M_r_C-) and spontaneous double positive (M_sp_C+) cell subpopulations.**

Each line represents an independent measurement. At least four independent measurements (n≥4) were taken for each of the four TNF-α and SAHA-responsive single mCherry positive (M_TS_C+) cell clones representing two vector integration sites. Two-tailed Mann-Whitney *U* test with 95% confidence level was used to test for statistical significance; **P*<0.05; ****P*<0.001.

**
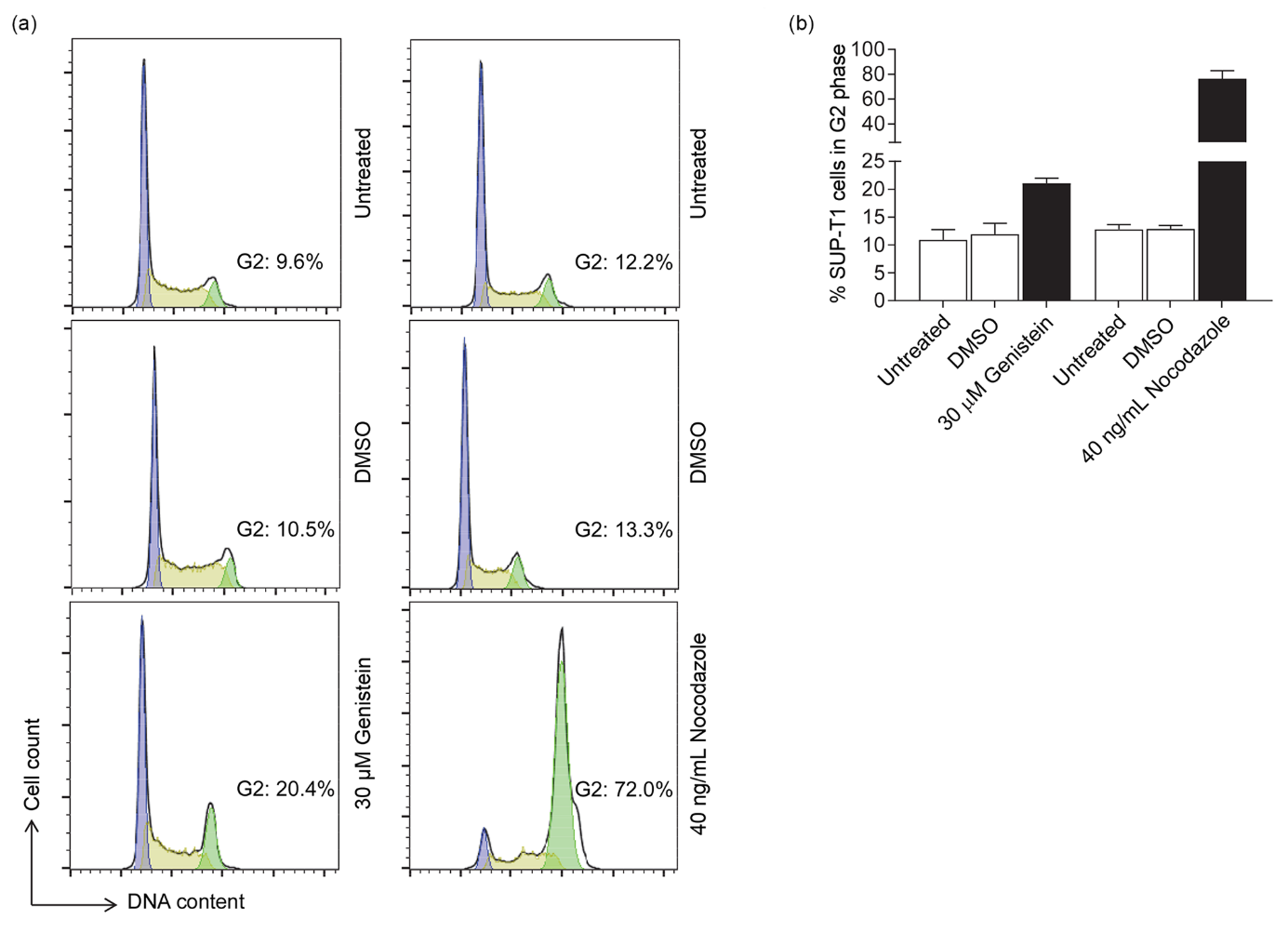
**

**Supplementary Fig. S5: Effects of genistein and nocodazole on SUP-T1 cells.**

(a) Representative cell cycle profiles of SUP-T1 cells upon treatment with genistein (left panel) and nocodazole (right panel) in comparison to untreated control (top panel) and equally diluted DMSO control (middle panel). The percentages of SUP-T1 cells in the G2 phase of the cell cycle are indicated. (b) Bar graph depicts the means of two independent measurements (n=2) (heights of the bars) and standard error means (error bars).

**Supplementary Table S1. Mutations in the integrated Cerulean cassettes of TNF-α and SAHA-responsive single mCherry positive (M_TS_C+) cell clones.**

| **Cell clone** | **Mutation** | | **DNA motif/protein domain** |
| --- | --- | --- | --- |
| M_TS_C_1_+3 | None | Not applicable | Not applicable |
| M_TS_C_1_+8 | Tat | G2180A (G5959A) | Tat activation domain (G44S) |
| M_TS_C_1_+12 | 5' LTR | A475T (A475T) | TAR 3-nucleotide bulge |
| M_TS_C_1_+16 | Tat | G2181A (G5960A) | Tat activation domain (G44D) |
| M_TS_C_2_+11 | 5' LTR | C280T (C280T) | 5' LTR U3 |
|  | RRE | G1387A (G7835A) | RRE stem IIB |
|  | Tat/Rev intron | G1666A (G8114A) | Not applicable |
|  | Tat | C2246T (C6025T) | 18 nucleotides from the 3' end of Tat exon 1 (Q66Stop) |
| M_TS_C_2_+13 | 5' LTR | C110T (C110T) | 5' LTR U3 |
|  |  | G243A (G243A) | 5' LTR U3 |
|  |  | C439T (C439T) | 5' LTR U3 |
|  |  | G695A (G695A) | 5' LTR U5 |
|  |  | G727A (G727A) | Stem loop 1 |
|  |  | G752A (G752A) | Stem loop 2 |
|  |  | G754A (G754A) | Stem loop 2 |
|  |  | G769A (G769A) | Stem loop 3/Ψ |
|  |  | C792A (C792A) | SalI restriction site |
|  | Tat/Rev intron | T1860C (T8308C) | Not applicable |
|  |  | G1920A (G8368A) | Not applicable |
| M_TS_C_2_+15 | None | Not applicable | Not applicable |

Nucleotide numbering is of LTatC[M] with the corresponding numbering in HIV-1_NL4-3_ in parenthesis. Amino acid numbering is shown in the ‘DNA motif/protein domain’ column. Subscripts 1 and 2 indicate two independent transduction and sorting experiments from which the clones were derived.

**Supplementary Table S2a: Genes with a significantly higher expression level in spontaneous double positive (M_sp_C+) cell subpopulations compared to their counterparts that remained single mCherry positive (M_r_C-).**

| **Gene** | **Gene type** | **\|log_2_ratio\|** | ***P* value** | **Involvement in** | |
| --- | --- | --- | --- | --- | --- |
|  |  |  |  | **Cell cycle** | **HIV-1 replication** |
| *BZRAP1* | Protein coding | 1.376 | 1.12E-14 |  |  |
|  |  | 0.8946 | 1.77E-02 |  |  |
| *CTB-119C2.1* | Antisense RNA | 0.7186 | 5.76E-03 |  |  |
|  |  | 1.736 | 2.15E-05 |  |  |
| *DHRS2* | Protein coding | 1.049 | 4.18E-04 | Possibly^1^ |  |
|  |  | 1.328 | 2.52E-04 |  |  |
| *EGR1* | Protein coding | 0.6368 | 6.36E-03 | Yes(+)^2-5^ | Yes^6-10^ |
|  |  | 0.9895 | 1.34E-02 |  |  |
| *FOSB* | Protein coding | 2.38 | 1.71E-21 | Yes(+)^11^ |  |
|  |  | 1.378 | 3.35E-03 |  |  |
| *GPR17* | Protein coding | 1.973 | 4.08E-04 |  |  |
|  |  | 2.127 | 3.31E-04 |  |  |
| *MT-ND2* | Protein coding | 0.7098 | 5.37E-03 |  |  |
|  |  | 1.136 | 1.25E-02 |  |  |
| *MT-TI* | ncRNA | 2.102 | 1.26E-30 |  |  |
|  |  | 3.401 | 2.63E-22 |  |  |
| *NEAT1* | lncRNA | 2.155 | 3.47E-24 | Yes(+)^12^ | Yes^13^ |
|  |  | 1.151 | 1.76E-02 |  |  |
| *PILRA* | Protein coding | 0.8354 | 6.96E-05 |  |  |
|  |  | 1.107 | 1.69E-02 |  |  |
| *RP11-54O7.17* | lincRNA | 1.371 | 2.06E-06 |  |  |
|  |  | 1.921 | 1.14E-05 |  |  |
| *RP11-196G18.22* | lncRNA | 1.377 | 3.02E-07 |  |  |
|  |  | 1.427 | 2.80E-06 |  |  |
| *RP11-196G18.24* | lincRNA | 0.7146 | 1.61E-02 |  |  |
|  |  | 1.675 | 1.70E-04 |  |  |
| *SLX1A-SULT1A3* | ncRNA | 0.6932 | 9.60E-03 |  |  |
|  |  | 1.505 | 4.83E-03 |  |  |
| *SNORD3B-1* | snoRNA | 0.9134 | 1.31E-03 |  |  |
|  |  | 1.565 | 1.00E-02 |  |  |
| *snoU2-30* | snoRNA | 0.785 | 3.43E-03 |  |  |
|  |  | 1.683 | 3.38E-03 |  |  |
| *SULT1A3* | Protein coding | 0.7184 | 1.61E-02 |  | Possibly^14,15^ |
|  |  | 2.049 | 9.66E-06 |  |  |
| *Y_RNA* | Y RNA | 1.686 | 1.15E-09 | Possibly^16,17^ |  |
|  |  | 0.8761 | 1.22E-02 |  |  |

|log2ratio| and *P* values for the two groups of M_TS_C+ cell clones are shown. + indicates positive effects on the cell cycle. ncRNA: non-coding RNA; lncRNA: long non-coding RNA; lincRNA: large intergenic non-coding RNA; snoRNA: small nucleolar RNA.

**Supplementary Table S2b: Genes with a significantly higher expression level in cell subpopulations that remained single mCherry positive (M_r_C-) compared to their spontaneous double positive counterparts (M_sp_C+).**

| **Gene** | **Gene type** | **\|log_2_ratio\|** | ***P* value** | **Involvement in** | |
| --- | --- | --- | --- | --- | --- |
|  |  |  |  | **Cell cycle** | **HIV-1 replication** |
| *AL355075.1* | miRNA | 1.065 | 2.96E-03 |  |  |
|  |  | 2.259 | 3.60E-06 |  |  |
| *CTD-2410N18.5* | Protein coding | 0.7013 | 1.46E-06 |  |  |
|  |  | 1.194 | 8.27E-04 |  |  |
| *MIR1248* | miRNA | 1.159 | 1.07E-06 |  | Possibly^18^ |
|  |  | 1.406 | 1.86E-02 |  |  |
| *MIR1291* | miRNA | 1.331 | 3.34E-08 | Yes(-)^19^ |  |
|  |  | 1.876 | 1.02E-05 |  |  |
| *MIR3655* | miRNA | 0.718 | 4.04E-04 |  |  |
|  |  | 0.8275 | 2.49E-03 |  |  |
| *MIR3917* | miRNA | 1.091 | 3.86E-07 |  |  |
|  |  | 0.9886 | 6.84E-04 |  |  |
| *MIR6516* | miRNA | 1.599 | 5.79E-10 |  |  |
|  |  | 1.354 | 3.36E-03 |  |  |
| *MORF4L1P1* | Pseudogene | 0.6869 | 6.07E-03 |  |  |
|  |  | 1.13 | 1.60E-04 |  |  |
| *RMRP* | Ribozyme | 0.9398 | 2.69E-07 | Yes(+)^20,21^ |  |
|  |  | 1.125 | 2.37E-03 |  |  |
| *RN7SK* | snRNA | 1.028 | 1.20E-03 | Yes(-)^22^ | Yes^23^ |
|  |  | 1.632 | 6.96E-03 |  |  |
| *RN7SKP255* | ncRNA | 1.01 | 7.37E-04 |  |  |
|  |  | 1.958 | 2.56E-06 |  |  |
| *RN7SL1* | SRP RNA | 0.891 | 5.41E-04 |  | Yes^24^ |
|  |  | 1.188 | 1.09E-02 |  |  |
| *RN7SL2* | SRP RNA | 0.9643 | 1.25E-04 |  | Yes^24^ |
|  |  | 1.434 | 2.39E-03 |  |  |
| *RNA5SP202* | rRNA | 1.211 | 1.66E-02 |  |  |
|  |  | 1.112 | 1.70E-02 |  |  |
| *RNU2-2P* | snRNA | 1.078 | 1.87E-05 |  |  |
|  |  | 0.9861 | 1.29E-04 |  |  |
| *RNU4-2* | snRNA | 1.399 | 1.00E-09 |  |  |
|  |  | 0.8982 | 3.71E-03 |  |  |
| *RNY1* | Y RNA | 1.439 | 1.28E-06 | Yes(+)^16,17^ | Yes^24^ |
|  |  | 1.725 | 1.11E-04 |  |  |
| *RNY3* | Y RNA | 1.824 | 1.47E-09 | Yes(+)^16,17^ | Yes^24^ |
|  |  | 1.422 | 4.93E-03 |  |  |
| *RP11-101E3.5* | Protein  coding | 0.6188 | 1.08E-03 |  |  |
|  |  | 0.6561 | 1.42E-02 |  |  |
| *RP11-575L7.8* | Antisence RNA | 0.9566 | 5.47E-03 |  |  |
|  |  | 1.424 | 4.07E-03 |  |  |

| *RP11-618G20.1* | Processed transcript | 0.6568 | 9.82E-04 |  |  |
| --- | --- | --- | --- | --- | --- |
|  |  | 0.7369 | 5.96E-03 |  |  |
| *SCARNA10* | scaRNA | 1.358 | 3.25E-16 |  |  |
|  |  | 2.15 | 2.04E-10 |  |  |
| *SCARNA12* | scaRNA | 0.9441 | 5.06E-06 |  |  |
|  |  | 1.606 | 4.18E-07 |  |  |
| *SCARNA16* | scaRNA | 1.709 | 9.95E-20 |  |  |
|  |  | 1.463 | 4.72E-04 |  |  |
| *SCARNA22* | scaRNA | 1.387 | 1.15E-12 |  |  |
|  |  | 1.358 | 8.17E-05 |  |  |
| *SCARNA5* | scaRNA | 1.616 | 3.59E-09 |  |  |
|  |  | 2.461 | 8.40E-07 |  |  |
| *SCARNA6* | scaRNA | 1.164 | 4.47E-08 |  |  |
|  |  | 2.401 | 3.63E-11 |  |  |
| *SNORA10* | snoRNA | 1.018 | 7.03E-05 |  |  |
|  |  | 1.197 | 8.70E-03 |  |  |
| *SNORA12* | snoRNA | 1.096 | 8.97E-06 |  |  |
|  |  | 1.826 | 3.03E-04 |  |  |
| *SNORA14B* | snoRNA | 1.267 | 7.64E-05 |  |  |
|  |  | 1.344 | 4.32E-03 |  |  |
| *SNORA20* | snoRNA | 1.806 | 1.98E-11 |  |  |
|  |  | 2.137 | 5.80E-05 |  |  |
| *SNORA21* | snoRNA | 1.666 | 5.95E-15 |  |  |
|  |  | 1.896 | 1.31E-06 |  |  |
| *SNORA22* | snoRNA | 2.618 | 9.26E-25 |  |  |
|  |  | 1.378 | 4.85E-03 |  |  |
| *SNORA23* | snoRNA | 1.925 | 3.23E-12 |  |  |
|  |  | 1.613 | 1.94E-04 |  |  |
| *SNORA24* | snoRNA | 1.66 | 2.85E-08 |  |  |
|  |  | 2.163 | 5.29E-08 |  |  |
| *SNORA26* | snoRNA | 1.166 | 4.04E-04 |  |  |
|  |  | 1.558 | 9.27E-04 |  |  |
| *SNORA27* | snoRNA | 0.8787 | 1.86E-03 |  |  |
|  |  | 1.076 | 1.97E-02 |  |  |
| *SNORA31* | snoRNA | 1.637 | 5.18E-10 |  |  |
|  |  | 2.007 | 4.94E-07 |  |  |
| *SNORA34* | snoRNA | 1.332 | 1.62E-08 | Yes(-)^19^ |  |
|  |  | 1.877 | 9.67E-06 |  |  |
| *SNORA37* | snoRNA | 0.9123 | 9.02E-04 |  |  |
|  |  | 0.8213 | 8.31E-03 |  |  |
| *SNORA38B* | snoRNA | 0.8618 | 1.97E-02 |  |  |
|  |  | 1.981 | 7.09E-05 |  |  |
| *SNORA44* | snoRNA | 1.384 | 9.65E-09 |  |  |
|  |  | 1.046 | 1.96E-02 |  |  |
| *SNORA48* | snoRNA | 1.766 | 8.80E-16 |  |  |
|  |  | 1.376 | 9.80E-04 |  |  |
| *SNORA49* | snoRNA | 1.59 | 1.80E-16 |  |  |
|  |  | 1.83 | 1.96E-08 |  |  |

| *SNORA54* | snoRNA | 1.272 | 1.74E-05 |  |  |
| --- | --- | --- | --- | --- | --- |
|  |  | 1.44 | 3.60E-03 |  |  |
| *SNORA62* | snoRNA | 1.607 | 1.71E-15 |  |  |
|  |  | 1.811 | 2.34E-06 |  |  |
| *SNORA63* | snoRNA | 1.234 | 2.24E-07 |  |  |
|  |  | 1.49 | 9.54E-05 |  |  |
| *SNORA68* | snoRNA | 1.446 | 3.06E-11 |  |  |
|  |  | 2.107 | 6.21E-06 |  |  |
| *SNORA71C* | snoRNA | 1.461 | 7.39E-09 |  |  |
|  |  | 2.219 | 1.26E-06 |  |  |
| *SNORA71D* | snoRNA | 1.603 | 8.35E-11 |  |  |
|  |  | 1.858 | 6.84E-05 |  |  |
| *SNORA73A* | snoRNA | 1.6 | 7.44E-15 |  |  |
|  |  | 1.531 | 4.66E-07 |  |  |
| *SNORA73B* | snoRNA | 1.882 | 1.21E-17 |  |  |
|  |  | 2.164 | 4.10E-11 |  |  |
| *SNORA79* | snoRNA | 1.034 | 1.02E-03 |  |  |
|  |  | 2.031 | 2.83E-05 |  |  |
| *SNORA7A* | snoRNA | 0.7537 | 4.60E-05 | Yes(+)^25^ |  |
|  |  | 0.8442 | 1.83E-02 |  |  |
| *SNORA81* | snoRNA | 1.118 | 1.93E-06 |  |  |
|  |  | 1.401 | 1.40E-02 |  |  |
| *SNORD10* | snoRNA | 1.828 | 1.85E-17 |  |  |
|  |  | 1.598 | 5.09E-08 |  |  |
| *SNORD104* | snoRNA | 0.5859 | 1.50E-02 |  |  |
|  |  | 1.103 | 1.77E-02 |  |  |
| *SNORD105B* | snoRNA | 0.8567 | 1.19E-02 |  |  |
|  |  | 1.588 | 1.71E-03 |  |  |
| *SNORD107* | snoRNA | 0.6313 | 1.53E-02 |  |  |
|  |  | 1.207 | 8.49E-03 |  |  |
| *SNORD111B* | snoRNA | 0.8842 | 2.42E-03 |  |  |
|  |  | 1.554 | 5.28E-04 |  |  |
| *SNORD116-1* | snoRNA | 0.7306 | 3.60E-03 |  |  |
|  |  | 1.266 | 1.52E-02 |  |  |
| *SNORD116-14* | snoRNA | 0.7032 | 1.79E-02 |  |  |
|  |  | 1.765 | 2.47E-04 |  |  |
| *SNORD116-15* | snoRNA | 1.48 | 3.26E-06 |  |  |
|  |  | 1.647 | 7.34E-04 |  |  |
| *SNORD116-16* | snoRNA | 1.613 | 1.07E-09 |  |  |
|  |  | 2.06 | 5.04E-06 |  |  |
| *SNORD116-2* | snoRNA | 1.344 | 2.32E-07 |  |  |
|  |  | 1.715 | 9.95E-04 |  |  |
| *SNORD116-22* | snoRNA | 1.334 | 5.01E-06 |  |  |
|  |  | 2.097 | 2.40E-05 |  |  |
| *SNORD116-23* | snoRNA | 1.245 | 1.47E-04 |  |  |
|  |  | 2.129 | 1.85E-05 |  |  |
| *SNORD116-24* | snoRNA | 1.95 | 1.06E-12 |  |  |
|  |  | 2.248 | 8.02E-07 |  |  |

| *SNORD116-6* | snoRNA | 0.8557 | 5.27E-03 |  |  |
| --- | --- | --- | --- | --- | --- |
|  |  | 1.343 | 3.37E-03 |  |  |
| *SNORD116-8* | snoRNA | 1.588 | 3.42E-09 |  |  |
|  |  | 2.801 | 5.61E-11 |  |  |
| *SNORD15A* | snoRNA | 1.521 | 2.93E-14 |  |  |
|  |  | 0.9395 | 1.41E-02 |  |  |
| *SNORD15B* | snoRNA | 2.445 | 1.82E-33 |  |  |
|  |  | 1.465 | 4.17E-08 |  |  |
| *SNORD17* | snoRNA | 1.782 | 3.80E-13 |  |  |
|  |  | 2.092 | 4.83E-10 |  |  |
| *SNORD26* | snoRNA | 0.9865 | 3.60E-06 |  |  |
|  |  | 2.22 | 2.68E-09 |  |  |
| *SNORD27* | snoRNA | 1.488 | 2.08E-05 | Possibly^26^ |  |
|  |  | 1.698 | 1.23E-05 |  |  |
| *SNORD3A* | snoRNA | 1.387 | 1.43E-26 |  |  |
|  |  | 0.9987 | 1.10E-03 |  |  |
| *SNORD42A* | snoRNA | 1.039 | 1.39E-03 |  |  |
|  |  | 1.897 | 1.08E-04 |  |  |
| *SNORD46* | snoRNA | 1.746 | 2.70E-09 |  |  |
|  |  | 1.752 | 1.46E-04 |  |  |
| *SNORD58A* | snoRNA | 1.185 | 6.53E-07 |  |  |
|  |  | 1.574 | 6.75E-05 |  |  |
| *SNORD58B* | snoRNA | 1.569 | 1.68E-09 |  |  |
|  |  | 1.109 | 4.20E-04 |  |  |
| *SNORD63* | snoRNA | 1.604 | 3.26E-09 |  |  |
|  |  | 1.326 | 4.69E-03 |  |  |
| *SNORD8* | snoRNA | 1.712 | 3.79E-14 |  |  |
|  |  | 1.775 | 3.27E-08 |  |  |
| *SNORD97* | snoRNA | 1.648 | 8.89E-13 |  |  |
|  |  | 2.563 | 1.06E-15 |  |  |
| *SNORD99* | snoRNA | 0.6244 | 9.39E-03 |  |  |
|  |  | 1.231 | 3.31E-03 |  |  |
| *TMEM106B* | Protein coding | 0.6064 | 2.92E-03 | Yes(+)^27^ |  |
|  |  | 0.7932 | 6.52E-03 |  |  |
| *YEATS4* | Protein coding | 0.6928 | 1.58E-06 | Yes(+)^28,29^ |  |
|  |  | 0.611 | 1.59E-02 |  |  |

|log2ratio| and *P* values for the two groups of M_TS_C+ cell clones are shown. + and - indicate positive and negative effects on the cell cycle, respectively. miRNA: microRNA; snRNA: small nuclear RNA; ncRNA: non-coding RNA; SRP RNA: signal recognition particle RNA; scaRNA: small cajal body-specific RNA; snoRNA: small nucleolar RNA.

**Supplementary Table 3: Genetic elements in LTatC[M].**

| **Genetic element** | **Function** | **Comment** |
| --- | --- | --- |
| HIV-1 5′ and 3′LTRs | (i) Targeting of HIV-1 integration sites.  (ii) Contain the HIV-1 promoter. |  |
| HIV-1 ψ and RRE | Packaging of LTatC[M] RNA genome. | HIV-1 Rev is encoded in the packaging plasmid psPAX2. |
| HIV-1 Tat | Efficient transcription from the HIV-1 promoter. |  |
| IRES | Co-expression of HIV-1 Tat and Cerulean. |  |
| Cerulean | Fluorescence reporter gene for HIV-1 promoter activity. |  |
| cHS4 and sMAR | (i) Prevent transcriptional interference between Cerulean and mCherry.  (ii) Prevent position-effect variegation silencing of mCherry. | Position and copy number are based on ^30^, ^31^, and ^32^. |
| TetO | Enhancement of mCherry expression when necessary. | Unnecessary for the present study, but might be useful when mCherry is silenced. |
| WPRE | Efficient expression of transgenes. |  |

LTR: long terminal repeat; ψ: packaging signal; RRE: Rev response element; Tat: transactivator of transcription; IRES: internal ribosome entry site; cHS4: chicken hypersensitive site 4 core; sMAR: synthetic matrix attachment region; TetO: Tet operator; WPRE: Woodchuck hepatitis virus post-transcriptional regulatory element.

**References**

1 van der Meijden, C. M. *et al.* Gene profiling of cell cycle progression through S-phase reveals sequential expression of genes required for DNA replication and nucleosome assembly. *Cancer Res.* **62**, 3233-3243 (2002).

2 Pritchard, M. T., Malinak, R. N. & Nagy, L. E. Early growth response (EGR)-1 is required for timely cell-cycle entry and progression in hepatocytes after acute carbon tetrachloride exposure in mice. *Am. J. Physiol. Gastrointest. Liver Physiol.* **300**, G1124-1131, doi:10.1152/ajpgi.00544.2010 (2011).

3 Molnar, G., Crozat, A. & Pardee, A. B. The immediate-early gene Egr-1 regulates the activity of the thymidine kinase promoter at the G0-to-G1 transition of the cell cycle. *Mol. Cell. Biol.* **14**, 5242-5248 (1994).

4 Min, I. M. *et al.* The transcription factor EGR1 controls both the proliferation and localization of hematopoietic stem cells. *Cell Stem Cell* **2**, 380-391, doi:10.1016/j.stem.2008.01.015 (2008).

5 Hallahan, D. E. *et al.* C-jun and Egr-1 participate in DNA synthesis and cell survival in response to ionizing radiation exposure. *J. Biol. Chem.* **270**, 30303-30309 (1995).

6 Krishnan, V. & Zeichner, S. L. Host cell gene expression during human immunodeficiency virus type 1 latency and reactivation and effects of targeting genes that are differentially expressed in viral latency. *J. Virol.* **78**, 9458-9473, doi:10.1128/jvi.78.17.9458-9473.2004 (2004).

7 van 't Wout, A. B. *et al.* Cellular gene expression upon human immunodeficiency virus type 1 infection of CD4(+)-T-cell lines. *J. Virol.* **77**, 1392-1402 (2003).

8 Dron, M. *et al.* Cloning of a long HIV-1 readthrough transcript and detection of an increased level of early growth response protein-1 (Egr-1) mRNA in chronically infected U937 cells. *Arch. Virol.* **144**, 19-28 (1999).

9 Fan, Y., Zou, W., Green, L. A., Kim, B. O. & He, J. J. Activation of Egr-1 expression in astrocytes by HIV-1 Tat: new insights into astrocyte-mediated Tat neurotoxicity. *J. Neuroimmune Pharmacol.* **6**, 121-129, doi:10.1007/s11481-010-9217-8 (2011).

10 Kim, N., Kukkonen, S., Gupta, S. & Aldovini, A. Association of Tat with promoters of PTEN and PP2A subunits is key to transcriptional activation of apoptotic pathways in HIV-infected CD4+ T cells. *PLoS Pathog.* **6**, e1001103, doi:10.1371/journal.ppat.1001103 (2010).

11 Brown, J. R. *et al.* Fos family members induce cell cycle entry by activating cyclin D1. *Mol. Cell. Biol.* **18**, 5609-5619 (1998).

12 Li, Z. *et al.* Overexpression of long noncoding RNA, NEAT1 promotes cell proliferation, invasion and migration in endometrial endometrioid adenocarcinoma. *Biomed. Pharmacother.* **84**, 244-251, doi:10.1016/j.biopha.2016.09.008 (2016).

13 Zhang, Q., Chen, C. Y., Yedavalli, V. S. & Jeang, K. T. NEAT1 long noncoding RNA and paraspeckle bodies modulate HIV-1 posttranscriptional expression. *MBio* **4**, e00596-00512, doi:10.1128/mBio.00596-12 (2013).

14 Bruce, J. W., Ahlquist, P. & Young, J. A. The host cell sulfonation pathway contributes to retroviral infection at a step coincident with provirus establishment. *PLoS Pathog.* **4**, e1000207, doi:10.1371/journal.ppat.1000207 (2008).

15 Murry, J. P. *et al.* Sulfonation pathway inhibitors block reactivation of latent HIV-1. *Virology* **471-473**, 1-12, doi:10.1016/j.virol.2014.08.016 (2014).

16 Christov, C. P., Gardiner, T. J., Szuts, D. & Krude, T. Functional requirement of noncoding Y RNAs for human chromosomal DNA replication. *Mol. Cell. Biol.* **26**, 6993-7004, doi:10.1128/mcb.01060-06 (2006).

17 Christov, C. P., Trivier, E. & Krude, T. Noncoding human Y RNAs are overexpressed in tumours and required for cell proliferation. *Br. J. Cancer* **98**, 981-988, doi:10.1038/sj.bjc.6604254 (2008).

18 Chang, S. T. *et al.* Next-generation sequencing of small RNAs from HIV-infected cells identifies phased microrna expression patterns and candidate novel microRNAs differentially expressed upon infection. *MBio* **4**, e00549-00512, doi:10.1128/mBio.00549-12 (2013).

19 Tu, M. J., Pan, Y. Z., Qiu, J. X., Kim, E. J. & Yu, A. M. MicroRNA-1291 targets the FOXA2-AGR2 pathway to suppress pancreatic cancer cell proliferation and tumorigenesis. *Oncotarget* **7**, 45547-45561, doi:10.18632/oncotarget.9999 (2016).

20 Gill, T., Cai, T., Aulds, J., Wierzbicki, S. & Schmitt, M. E. RNase MRP cleaves the CLB2 mRNA to promote cell cycle progression: novel method of mRNA degradation. *Mol. Cell. Biol.* **24**, 945-953 (2004).

21 Thiel, C. T. *et al.* Severely incapacitating mutations in patients with extreme short stature identify RNA-processing endoribonuclease RMRP as an essential cell growth regulator. *Am. J. Hum. Genet.* **77**, 795-806, doi:10.1086/497708 (2005).

22 Eilebrecht, S. *et al.* 7SK small nuclear RNA directly affects HMGA1 function in transcription regulation. *Nucleic Acids Res.* **39**, 2057-2072, doi:10.1093/nar/gkq1153 (2011).

23 Karn, J. & Stoltzfus, C. M. Transcriptional and posttranscriptional regulation of HIV-1 gene expression. *Cold Spring Harb. Perspect. Med.* **2**, a006916, doi:10.1101/cshperspect.a006916 (2012).

24 Tian, C., Wang, T., Zhang, W. & Yu, X. F. Virion packaging determinants and reverse transcription of SRP RNA in HIV-1 particles. *Nucleic Acids Res.* **35**, 7288-7302, doi:10.1093/nar/gkm816 (2007).

25 Zhang, Y. *et al.* H/ACA Box Small Nucleolar RNA 7A Promotes the Self-Renewal of Human Umbilical Cord Mesenchymal Stem Cells. *Stem Cells* **35**, 222-235, doi:10.1002/stem.2490 (2017).

26 Falaleeva, M. *et al.* Dual function of C/D box small nucleolar RNAs in rRNA modification and alternative pre-mRNA splicing. *Proc. Natl. Acad. Sci. U. S. A.* **113**, E1625-1634, doi:10.1073/pnas.1519292113 (2016).

27 Brady, O. A., Zheng, Y., Murphy, K., Huang, M. & Hu, F. The frontotemporal lobar degeneration risk factor, TMEM106B, regulates lysosomal morphology and function. *Hum. Mol. Genet.* **22**, 685-695, doi:10.1093/hmg/dds475 (2013).

28 Tao, K., Yang, J., Hu, Y. & Deng, A. Knockdown of YEATS4 inhibits colorectal cancer cell proliferation and induces apoptosis. *Am J Transl Res* **7**, 616-623 (2015).

29 Pikor, L. A. *et al.* YEATS4 is a novel oncogene amplified in non-small cell lung cancer that regulates the p53 pathway. *Cancer Res.* **73**, 7301-7312, doi:10.1158/0008-5472.can-13-1897 (2013).

30 Tian, J. & Andreadis, S. T. Independent and high-level dual-gene expression in adult stem-progenitor cells from a single lentiviral vector. *Gene Ther* **16**, 874-884, doi:10.1038/gt.2009.46 (2009).

31 Chung, J. H., Bell, A. C. & Felsenfeld, G. Characterization of the chicken beta-globin insulator. *Proc Natl Acad Sci U S A* **94**, 575-580 (1997).

32 Recillas-Targa, F. *et al.* Position-effect protection and enhancer blocking by the chicken beta-globin insulator are separable activities. *Proc Natl Acad Sci U S A* **99**, 6883-6888, doi:10.1073/pnas.102179399 (2002).
